# Insights from multi-omics integration into seed germination of *Taxus chinensis* var *mairei*

**DOI:** 10.1038/s42003-023-05307-x

**Published:** 2023-09-11

**Authors:** Lulu Chen, Liang Qin, Yawen Zhang, Hualei Xu, Yufen Bu, Ran Wu, Haiqiang Liu, Qichen Hao, Hao Hu, Yijun Zhou, Jinchao Feng, Yanping Jing, Jun Han, Xiaodong Wang

**Affiliations:** 1 College of Life and Environmental Sciences, Centre for Imaging & Systems Biology, Minzu University of China, 100081 Beijing, China; 2 National Engineering Research Center of Tree Breeding and Ecological Restoration, College of Biological Sciences and Biotechnology, Beijing Forestry University, 100083 Beijing, China; 3 Key Laboratory of Mass Spectrometry Imaging and Metabolomics (Minzu University of China), State Ethnic Affairs Commission, 100081 Beijing, China; 4 Key Laboratory of Genetics and Breeding in Forest Trees and Ornamental Plants, Ministry of Education, College of Biological Sciences and Biotechnology, Beijing Forestry University, 100083 Beijing, China; 5 Genome British Columbia Proteomics Centre, University of Victoria, Victoria, BC V8Z 7X8 Canada; 6 Division of Medical Sciences, University of Victoria, Victoria, BC V8P 5C2 Canada

**Keywords:** Plant development, Plant hormones, Secondary metabolism

## Abstract

The transition from deep dormancy to seed germination is essential for the life cycle of plants, but how this process occurs in the gymnosperm Chinese yew (*Taxus chinensis* var *mairei*), the natural source of the anticancer drug paclitaxel, remains unclear. Herein, we analyse the transcriptome, proteome, spatial metabolome, and spatial lipidome of the Chinese yew and present the multi-omics profiles of dormant and germinating seeds. Our results show that abscisic acid and gibberellic acid 12 homoeostasis is closely associated with gene transcription and protein translation, and the balance between these phytohormones thereby determines if seeds remain dormant or germinate. We find that an energy supply of carbohydrates from glycolysis and the TCA cycle feed into the pentose phosphate pathway during seed germination, and energy supplied from lipids are mainly derived from the lipolysis of triacylglycerols. Using mass spectrometry imaging, we demonstrate that the spatial distribution of plant hormones and phospholipids has a remarkable influence on embryo development. We also provide an atlas of the spatial distribution of paclitaxel C in Chinese yew seeds for the first time. The data from this study enable exploration of the germination mechanism of Chinese yew seeds across several omics levels.

## Abstract (web summary)

Transcriptome, proteome, spatial metabolome, and spatial lipidome analyses provide insight into the mechanisms that regulate seed germination in the gymnosperm *Taxus chinensis var mairei* (Chinese yew).

## Introduction

Chinese yew (*Taxus chinensis* var *mairei*) is an endangered well-known Tertiary relic species endemic to China and mainly distributed in the Yangtze River basin. It is a natural source of paclitaxel (trademark Taxol), which is an important natural anti-cancer, antineoplastic, or cytotoxic chemotherapy drug^1^. Chinese yew populations have declined significantly because of overexploitation, slow growth, long seed dormancy, and poor seed germination. Chinese yew has been overexploited because of its outstanding medicinal properties, considerably increasing its extinction risk^2^. Although extensive studies have been conducted on seed dispersal or chemical surveys, information on the dynamic characteristics of seed dormancy and germination in Chinese yew is limited^3,4^.

Seed dormancy is defined as the failure of an intact viable seed to complete germination under any combination of normal physical environmental factors (temperature, light/dark, and water). According to the classification system proposed by Baskin, seeds with an underdeveloped embryo at dispersal are morphologically dormant (MD)^5^. If an additional physiological block inhibits germination, the seeds have morphophysiological dormancy (MPD). Chinese yew seeds are morphophysiologically dormant with a rigid testa, implying that the obstacles of testa, underdeveloped embryo, and physiological late maturation cause Chinese yew seeds dormancy^6^. Several theories on the regulatory mechanism of seed dormancy and germination have been developed, including plant hormonal regulation, phytochrome induction, Ca^2+^ signal fluctuation, hydration-dependent protein phase separation, and gene expression regulation^7–10^. Plant hormones (such as antagonistic metabolites abscisic acid and gibberellic acid) are closely related to gene transcription, protein expression, and chromatin remodelling, thereby regulating seed dormancy and germination^11,12^. The complete network of hormone-mediated seed dormancy and germination has been highlighted in *Arabidopsis* and many agricultural crops, such as rice, wheat, and soybean, but is rarely reported in Chinese yew^13–16^.

Chromosome-level *Taxus chinensis* var. *mairei* genome was sequenced in 2021, and a unique physical and functional grouping of *CYP725A*s (cytochrome P450) for paclitaxel biosynthesis was discovered at the genome level^17,18^. In contrast, the transcriptome, proteome, and metabolome of Chinese yew seeds have not been comprehensively characterised. We used RNA sequencing (RNA-seq) and state-of-the-art mass spectrometry to provide the first, to our knowledge, integrated transcriptomic, proteomic, spatial metabolomic, and spatial lipidomic atlas of Chinese yew seeds. We provide multiple lines of evidence that Chinese yew seed germination is activated by a combination of gene transcription, protein translation, and metabolic homoeostasis, especially by plant hormone regulation. Specifically, our spatial metabolomic and lipidomic results provide new insights into seed germination, showing that the spatial distribution of metabolites and lipids notably influences embryo development. These results contribute to our understanding of Chinese yew seed germination mechanisms and provide comprehensive insights into plant research across multi-omics levels.

## Results

### Multi-omics dataset of Chinese yew seeds

**Fig. 1:**
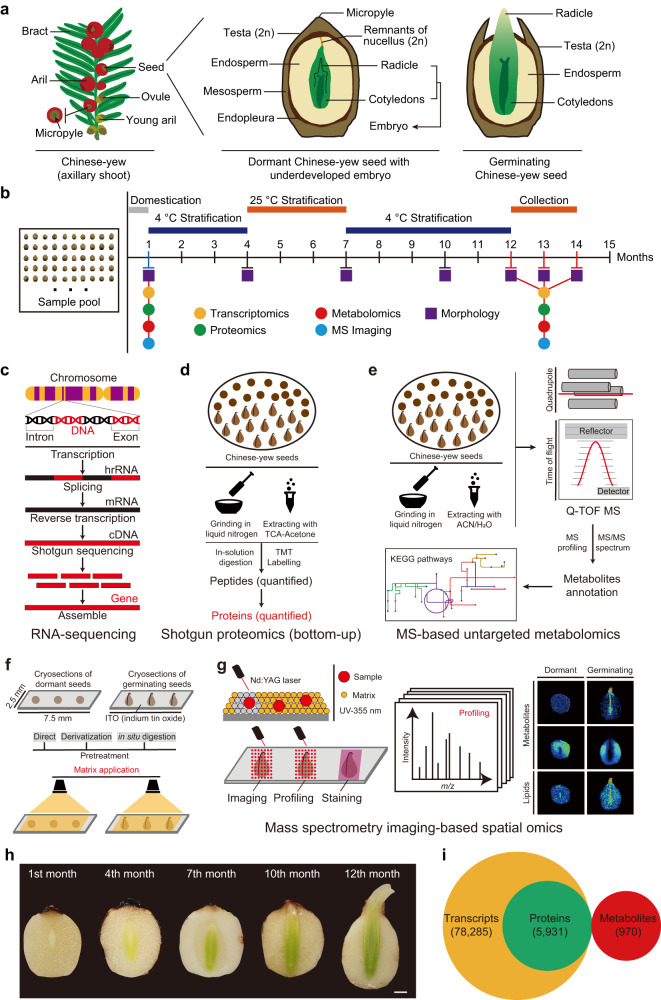
Overview of experimental design and multi-omics dataset. **a** Schematic of dormant and germinating Chinese yew seeds. **b** Sampling strategy. The study yielded multi-omics data from dormant and germinating seeds (every three month). **c**–**e** RNA sequencing-based transcriptomics, shotgun proteomics, and MS-based untargeted metabolomics experimental workflow of Chinese yew seed. **f**, **g** Mass spectrometry imaging-based spatial metabolomics and lipidomics experimental workflow of Chinese yew seed. **h** Optical micrographs of Chinese yew seeds. Dormant Chinese yew seeds (1st month) and germinating Chinese yew seeds (12th month) were used for multi-omics study. Scale bar, 100 µm. **i** Total number of identified transcripts, proteins, and metabolites in dormant and germinating seeds.

Seed germination is a complex physiological process that begins with the absorption of water and ends when the radicle breaks through a rigid testa (Fig. 1a). To comprehensively understand how seed germination is influenced or regulated by endogenous molecules, we developed a multi-omics method based on the transcriptome, proteome, metabolome, and spatial lipidome of Chinese yew seeds (Fig. 1b), conducted using RNA-seq, bottom–up quantitative proteomics, MS-based untargeted metabolomics, and mass spectrometry imaging techniques, respectively (Fig. 1c–g). In addition, morphological observations of dormant and germinating seeds confirmed the morphophysiological dormancy state of Chinese yew seeds (Fig. 1h). Using a reproducible biochemical and analytical approach, we generated a dataset covering 78,285 annotated transcripts, 5931 detected proteins, and 964 identified metabolites in Chinese yew seeds, making this study one of the most comprehensive Chinese yew seed transcriptome, proteome, and metabolome published to date (Fig. 1i).

### Gene transcription and protein translation during seed germination

Using RNA-seq, the transcriptomic data corroborated a substantial number of unigenes (110,996) and CDS (61,775) based on the detection of raw reads (Supplementary Fig. 1a). A total of 78,285 unigenes were annotated using seven common databases (Supplementary Fig. 1b and Supplementary Data 1–3). Among these annotated genes, 1,940 putative transcription factor encoding genes belonging to 59 major transcription factors (TFs) families were analysed to further investigate the transcriptional events of Chinese yew seed germination (Supplementary Fig. 1c and Supplementary Data 4). Statistical analysis identified 42,728 differentially expressed genes (DEGs) between dormant and germinating Chinese yew seeds (Supplementary Fig. 1d–f and Supplementary Data 5). Specifically, 20,845 genes were highly expressed in dormant seeds, and 21,883 genes were highly expressed in germinating seeds (Supplementary Fig. 1f and Supplementary Data 6). Furthermore, these annotated DEGs were assigned to functional categories by Gene Ontology (GO) enrichment analysis, including ‘integral component of membrane’ (7601 genes), ‘carbohydrate metabolic process’ (644 genes), ‘DNA-binding transcription factor activity’ (480 genes), and ‘paclitaxel biosynthetic process’ (133 genes) (Supplementary Fig. 1g, h and Supplementary Data 7). To verify some of the changes in mRNA that we detected by transcriptomics, we also examined the levels of selected transcripts by quantitative PCR with reverse transcription (RT-qRCR) (Supplementary Fig. 2).

**Fig. 2:**
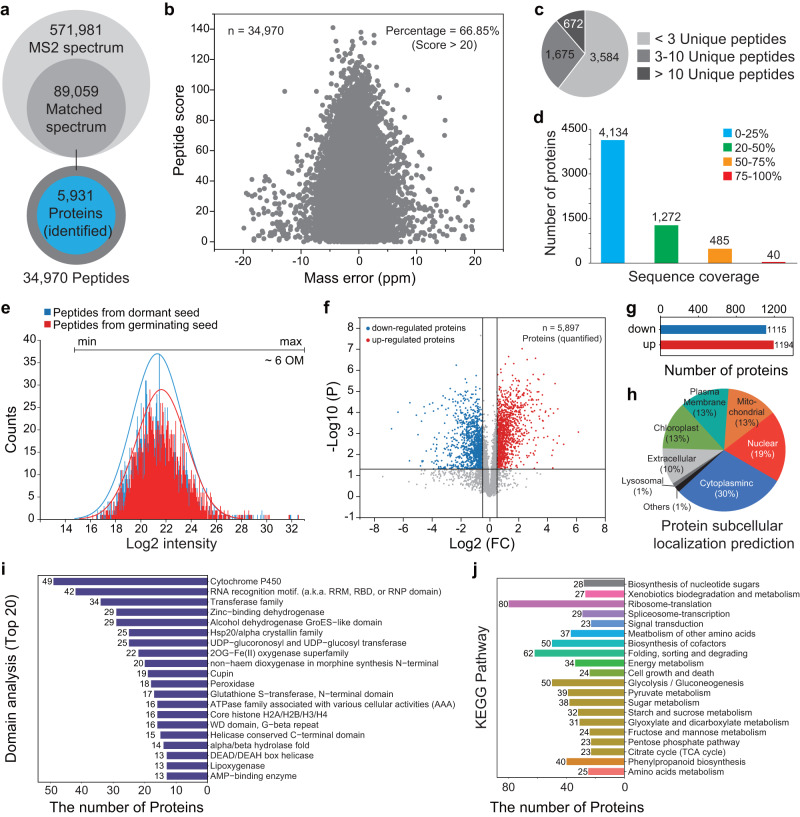
Descriptive analysis of the proteome of Chinese yew seeds. **a** Total number of MS/MS spectra acquired by LC-MS/MS, matched peptides, and identified proteins. **b** Scatter plot of peptide mass errors against peptide scores (*n* = 34,970). **c** Pie charts showing the percentage of proteins identified by <3, 3–10, or >10 peptides restricted to unique peptides only. **d** Number of proteins with different proportions of sequence coverage. **e** Distribution of the dynamic range of peptide abundance. OM (orders of magnitude). The peptide abundance spans six orders of magnitude. **f**, **g** Stage-specific proteins between dormant and germinating seeds. Upregulated and downregulated proteins denote more protein abundant in germinating and dormant seeds, respectively. Significant difference in proteins selected by volcano plot with fold change (FC > 2) and *P* values (*P* < 0.05). **h** Pie charts showing the predicted subcellular localisation of differentially expressed proteins. **i** Domain analysis of the differentially expressed proteins. Protein sequences were searched using InterProScan software to identify protein domain signatures from the InterPro member database Pfam. **j** KEGG enrichment analysis of differentially expressed proteins between dormant and germinating seeds.

Considering that translation is a crucial step contributing to gene expression, we further compared the proteome of dormant and germinating Chinese yew seeds. Using mass spectrometry, the proteomic data corroborated a substantial number of peptides (34,970) based on the 571,981 acquired MS/MS spectra of tryptic peptides from proteins extracted from Chinese yew seeds (Fig. 2a, b and Supplementary Data 8). In addition, mass spectrometry data enabled the detection of unique peptides for 5,931 proteins by searching against a self-built database (Fig. 2c, d and Supplementary Data 9). The dynamic range of peptide expression spanned six orders of magnitude (Fig. 2e). Notably, a strong variation in quantitative gene expression between dormant and germinating seeds was evident at the protein level (Fig. 2f, g and Supplementary data 10). 2,309 differentially expressed proteins were further analysed by subcellular localisation and Kyoto Encyclopedia of Genes and Genomes (KEGG) metabolic pathway prediction (Fig. 2h–j and Supplementary Data 11). As a result, a majority of differentially expressed proteins were involved in the ‘translation’, ‘folding, sorting and degrading’, ‘amino acids metabolism’, ‘glycolysis’, ‘tricarboxylic acid (TCA) cycle’, ‘energy metabolism’, and ‘metabolic pathway’ (Fig. 2j).

### Variation of mRNA and protein expression during Chinese yew seed germination

**Fig. 3:**
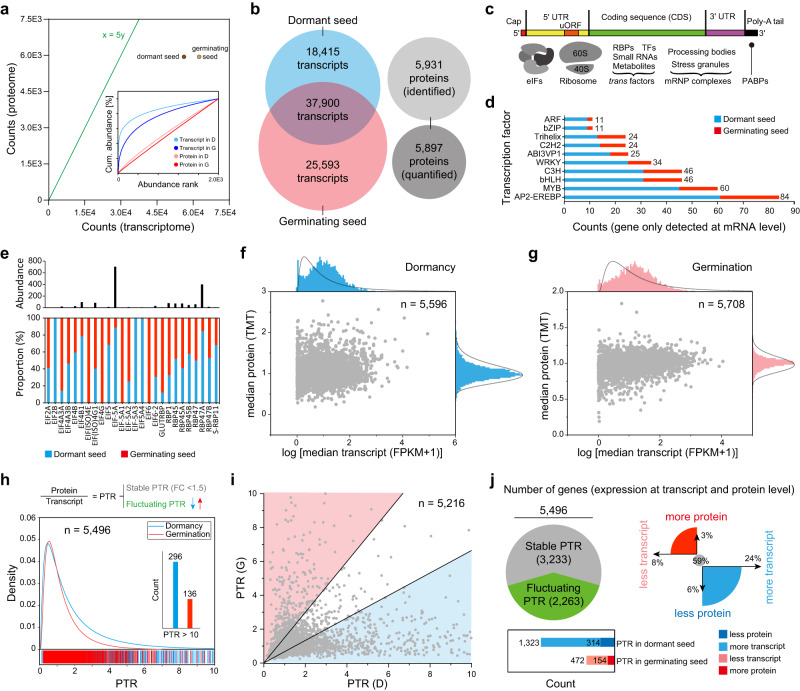
Gene expression at transcript and protein levels. **a**, **b** Total number of transcripts plotted against the total number of proteins detected in dormant and germinating seeds. Inset of (**a**) shows cumulative abundance plots of intensity-ranked (top 2000) identifications of transcripts and proteins for representative seeds. **c** Factors that influence mRNA translation. PABPs, phosphorylation of poly-A-binding proteins; eIFs, eukaryotic initiation factors; RBPs, RNA-binding proteins; TFs, transcription factors. **d** Number of TFs (top 10, only detected at mRNA level) in dormant and germinating seeds. **e** Top, summed absolute protein abundance of selected eIFs and RBPs. Bottom, protein abundance proportion of selected eIFs and RBPs between dormant and germinating seeds. **f**, **g** Scatter plot of protein-versus-transcript abundance in dormant and germinating seeds, respectively. **h** Top, Schematic of the steps in which modulation of the ratio of protein-to-mRNA (PTR) may occur. Bottom, PTR distribution of genes for core dataset (*n* = 5496). **i** Scatter plot of gene PTR value in dormant seeds against gene PTR value in germinating seeds. Fold change <1.5 was used to differentiate between stable and fluctuating PTR. **j** Analysis of the proportion of genes with stable or fluctuating PTRs.

Changes in gene expression are the core of most biological processes, particularly seed germination. The variation in transcripts (amount and abundance) was stronger between dormant and germinating Chinese yew seeds (Fig. 3a, b). Furthermore, only 10% of transcripts were translated into proteins. The actual translation efficiency in Chinese yew seeds is likely to be considerably higher as technical factors are limited (such as insufficient sequence coverage). The critical factors affecting translation included RNA structure, protein-RNA interactions, and ribosome occupancy (Fig. 3c). The number of transcription factors, only detected at the mRNA level, and the abundance of key eukaryotic initiation factors (eIFs), such as eIF4B1 and eIF5A, as well as RNA-binding proteins (RBPs), such as RBP45 and RBP47, in dormant seeds were higher than those in germinating seeds, which explains the slightly higher translation efficiency of storage proteins in dormant seeds (Fig. 3d, e and Supplementary Data 5).

We further obtained 5,496 core genes at the mRNA and protein levels in Chinese yew seeds by statistical analysis (Fig. 3f–h and Supplementary Data 12). The protein-to-transcript (PTR) ratio of core genes was used to evaluate the degree of variation during seed germination (Fig. 3h)^19^. Specifically, 3,233 genes with stable PTR (fold change <1.5) were probably under similar regulation in dormant and germinating seeds. In contrast, 2,263 fluctuating PTRs of genes between dormant and germinating seeds indicated development stage-specific regulation at either the transcript or the protein level (Fig. 3i, j). The fluctuation of PTR might be due to the differences in the translation efficiency of a given transcript, transcript stability, and/or stability of the respective protein. Plant hormone-mediated signalling pathway-related genes, such as the gibberellin biosynthesis gene *GA20OX1* and gibberellin receptor GA INSENSITIVE DWARF1(GID1), showed high PTR ratio in germinating seeds, indicating that they are rapidly translated from stored mRNA during germination. Conversely, the energy metabolism (glycolysis)-related genes *PFK1* and *ACS* showed low protein signals and high transcript levels in germinating seeds, implying that they are stored in seeds to be readily available for germination.

### Variation of metabolite expression and entire metabolic pathways involved in seed germination

**Fig. 4:**
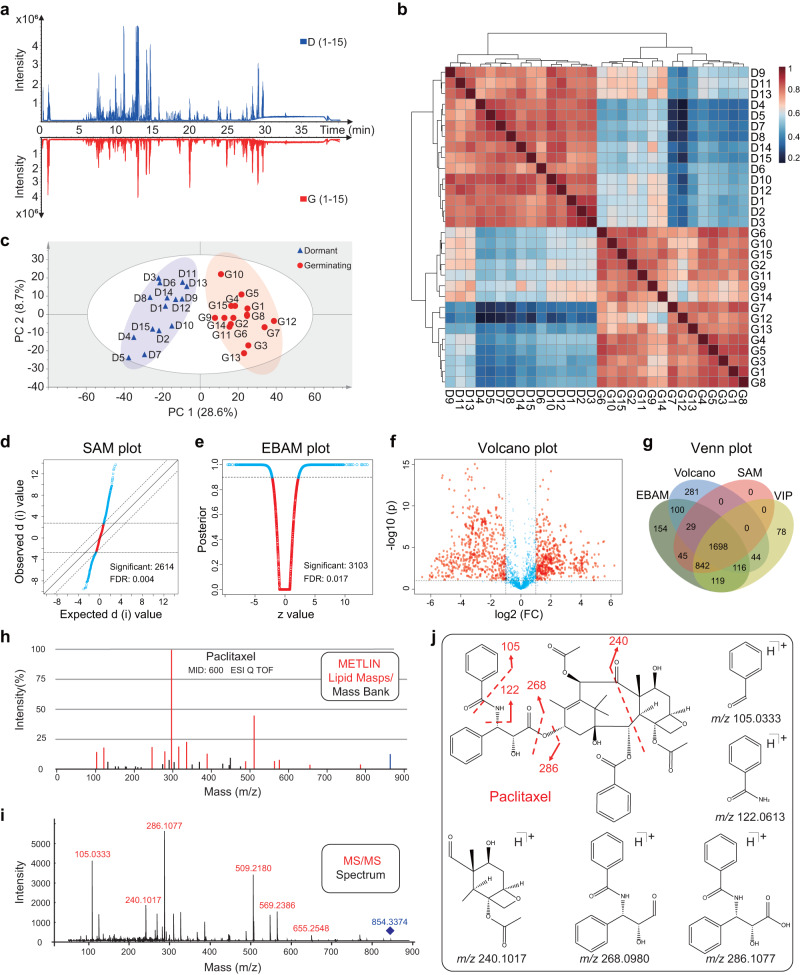
Descriptive analysis of the metabolome of Chinese yew seeds. **a** Comparison of base peak chromatograms acquired from dormant and germinating Chinese yew seeds by UPLC-QTOF MS/MS in the positive ion detection mode. D and G represent the dormancy (*n* = 15) and germination (*n* = 15) groups, respectively. **b** Pearson correlation cluster heatmap analysis of dormant and germinating Chinese yew seeds. Correlation values close to 1 represented a higher positive correlation, whereas values closer to 0 indicated no linear trend between the seed samples. **c** Principal component analysis (PCA) score plot of dormant and germinating Chinese yew seeds. Principal components 1 and 2 accounted for 37.3% of the total variance. **d** Significant difference in metabolites selected by significance analysis of metabolites (SAM). Blue circles represent features that exceed the specified threshold (false discovery rate (FDR) value < 0.01). **e** Significant difference metabolites selected by empirical Bayes analysis of metabolites (EBAM). Blue circles represent features that exceed the specified threshold (false discovery rate (FDR) value < 0.01). **f** Significant difference in metabolites selected by volcano plot with fold change (FC > 2) and *P* values (*P* < 0.05). **g** Venn diagram showing the overlap of significantly differentiated metabolites selected by variable importance value of the project (VIP, list from OPLS-DA analysis), EBAM, SAM, and volcano plot. A total of 1698 metabolites were identified as differentially expressed metabolites. **h**–**j** Illustration of the compound identification strategy using paclitaxel as an example. **h** denotes the MS/MS spectrum of paclitaxel in the METLIN database. **i** denotes the MS/MS spectrum of paclitaxel detected by UPLC-QTOF MS/MS. **j** Proposed structures of the fragment ions of paclitaxel by MS/MS.

Because genes with fluctuating PTR were enriched in metabolic pathways, and crucial plant hormones were assigned to small molecule metabolites, we further explored the global metabolome of Chinese yew seeds using an ultrahigh-performance liquid chromatography-tandem mass spectrometry (UPLC-MS/MS) untargeted metabolomics approach (Fig. 4a, Supplementary Data 13, and Supplementary Fig. 3). As expected, 1698 metabolites were proven to be differentially accumulated between dormant and germinating Chinese yew seeds using Pearson correlation analysis, principal component analysis, and multiple statistical analysis methods (Fig. 4b–g and Supplementary Data 14). By comparing the acquired MS/MS spectra of metabolites with those in the standard MS/MS libraries of the METLIN and LIPID MAPS databases, a total of 964 metabolites were identified, and a notable example of paclitaxel was exhibited (Fig. 4h–j and Supplementary Data 15 and 16). The cloud plot and enrichment analysis results showed that the amino acid content in germinating seeds was significantly higher than that in dormant seeds (Supplementary Fig. 4a–e). In contrast, TCA cycle-related metabolites, terpenoids, flavonoids, and alkaloids decreased in germinating seeds (Supplementary Fig. 4e and Supplementary Fig. 5).

**Fig. 5:**
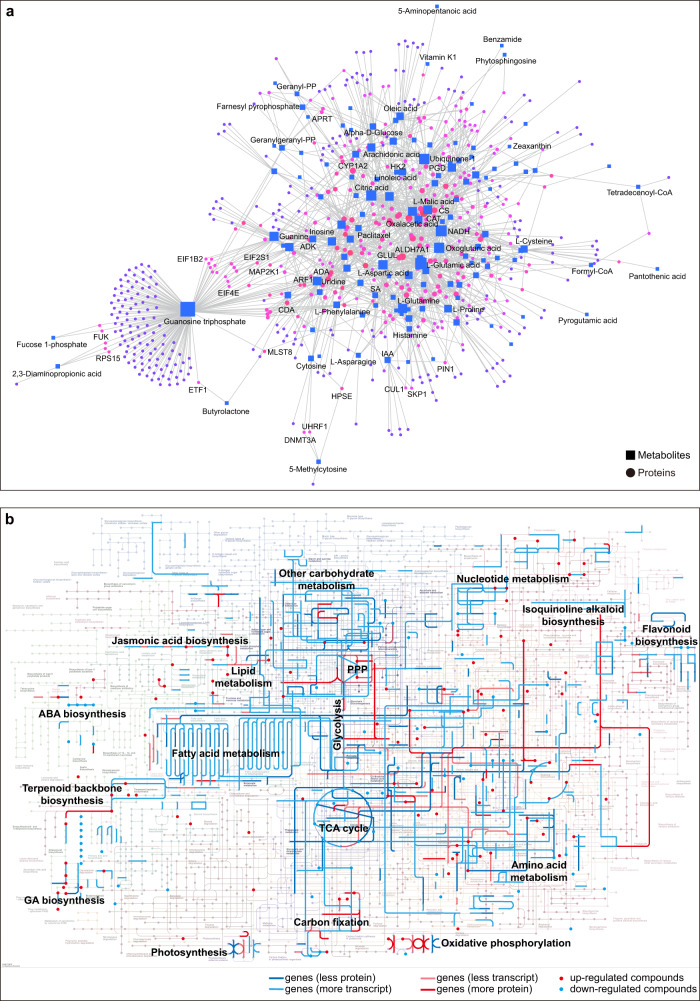
Joint analysis of Chinese yew seed transcriptome, proteome, and metabolome. **a** Interaction network of identified proteins and metabolites. The bigger the nodes, the stronger the correlation between the features. **b** Global metabolic pathways involved in Chinese yew seed germination. Genes with fluctuating PTR and differentially expressed metabolites were superimposed onto metabolic pathway maps based on the KEGG pathway. Red and blue colour denotes upregulated and downregulated expression of enzymes or metabolites (G vs D).

**Fig. 6:**
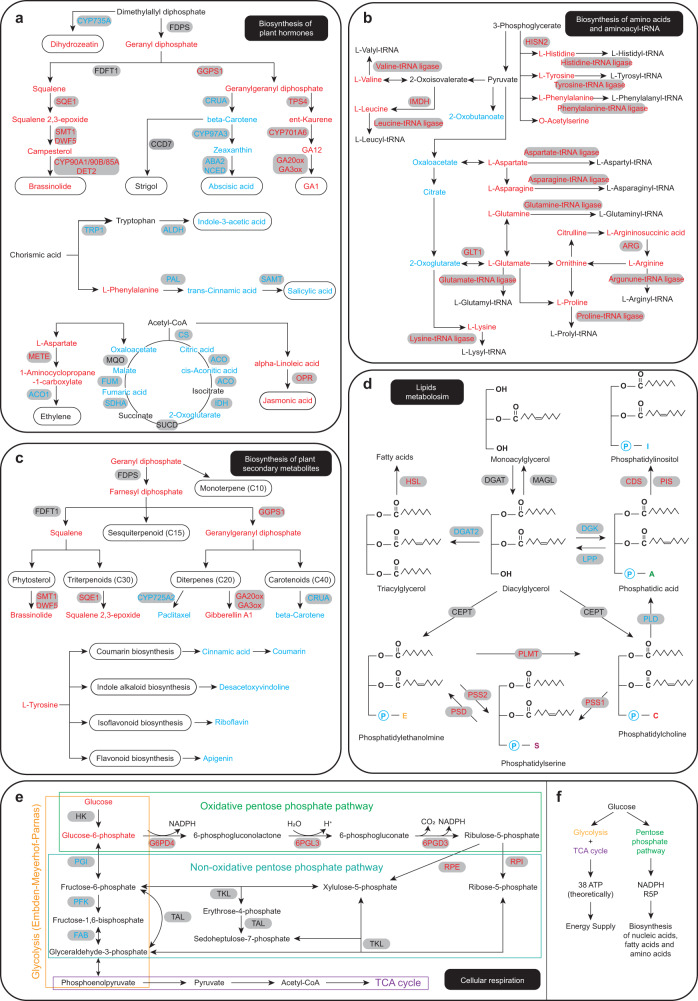
Summary diagram of specific metabolic pathways involved in Chinese yew seed germination. **a** Pathway of biosynthesis of plant hormones. **b** Biosynthesis pathway of amino acids and aminoacyl-tRNA. **c** Biosynthesis pathway of plant secondary metabolites. **d** Biosynthesis pathway of lipids metabolism. **e** Pathway of carbohydrates metabolism, including glycolysis, oxidative and non-oxidative pentose phosphate pathway. **f** Diagram summary of glucose metabolism. Red and blue colour denotes upregulated and downregulated expression of enzymes or metabolites (G vs D).

The relevance of crucial metabolites and proteins was evident when the metabolome and proteome were illustrated using the interaction network (Fig. 5a). Proteins associated with plant hormone biosynthesis, amino acid biosynthesis, energy metabolism, and secondary metabolism showed a strong interaction with corresponding metabolites, implying that these joint metabolic pathways were highly impacted during seed germination (Fig. 5a). To comprehensively understand the metabolic pathways involved in seed germination, we examined the transcriptomes, proteomes, and metabolome differences between dormant and germinating Chinese yew seeds (Fig. 5b). As described below, we followed the individual metabolites along with the transcript and protein abundances of the corresponding enzymes involved in plant hormone biosynthesis and signalling, energy supply (carbohydrates, lipids, and proteins), metabolic homoeostasis, and lipid metabolism (Fig. 6).

### Chinese yew seed germination undergoes plant hormones regulation

Similar to the previous analysis of the seed germination process, the endogenous abscisic acid (ABA) level was downregulated while gibberellin (GA_12_) content was upregulated in this study (Supplementary Fig. 4e), indicating that the mediation of ABA and GA balance is important for seed germination. ABA biosynthesis genes and signalling cascade (PYR/PYL/RCAR-PP2C-SnRKs-RAV) genes were decreased in germinating seeds (mRNA level), revealing that ABA signalling has a negative correlation with seed germination (Fig. 6a and Supplementary Figs. 2 and 6a). In contrast, high expression of GA biosynthesis genes and the GA receptor GID1 was found in germinating seeds (Fig. 6a and Supplementary Fig. 6b), demonstrating that GA signalling has a positive correlation with seed germination. As expected, DELLA, a negative regulator of GA signalling, showed a strong reduction in germinating seeds (protein level), further supporting the function of GA in breaking dormancy and stimulating Chinese yew seed germination described above (Supplementary Fig. 6b).

Earlier studies showed a close positive correlation between auxin and ABA and that ABA could repress radicle elongation during seed germination by potentiating auxin signalling^7^. Confirming our transcriptomic, proteomic, and metabolomic analyses, indole-3-acetic acid (IAA), auxin biosynthesis-related genes, auxin receptors, and auxin-responsive transcription factors ARF10 and ARF16 were significantly decreased in germinating seeds (Supplementary Figs. 2 and 6c), suggesting that auxin contents/signalling were, at least partially, associated with seed dormancy and germination. However, the mechanism by which auxin regulates seed dormancy in Chinese yew requires further study.

### Energy supply and metabolic homoeostasis regarding embryo development

To examine the energy demands of embryo development, we followed the alterations of metabolites and corresponding enzymes associated with amino acid biosynthesis and metabolism and glucose and lipid metabolism (Figs. 5b and 6b–f). Increased degradation of storage proteins was linked to substantial increases in the level of amino acids, consequently strongly increasing the biosynthesis of proteins required for embryo development (underpinned by increased amino acid-tRNA ligase) (Figs. 5 and 6b and Supplementary Fig. 4e). The degradation or catabolism of amino acids can also contribute to the energy state of plant cells under certain physiological conditions (such as carbon starvation), whereas amino acids are used for the biosynthesis of proteins during Chinese yew seed germination, which is underpinned by decreased amino acid metabolism-related enzymes (Fig. 5). Several secondary metabolites produced from amino acids, such as terpenoids and alkaloids, consequently showed a decreasing trend with corresponding mRNAs and proteins in germinating seed (Figs. 5 and 6c). In contrast to the accumulation of primary metabolites, declined secondary metabolites might, at least partially, represent metabolic homoeostasis during Chinese yew seed germination. Notably, as one of the secondary metabolites, paclitaxel (tetracyclic diterpene) and eight paclitaxel precursors, taxa-4(5),11(12)-diene, taxa-4(20),11(12)-dien-5α-ol, taxa-4(20),11(12)-dien-5α,13α-diol, 10-deacetyl-2-debenzoylbaccatin III, 10-deacetylbaccatin III, baccatin III, 3’-N-debenzoyl-2’-deoxytaxol, and 3’-N-debenzoyltaxol, were predominantly accumulated in dormant Chinese yew seeds; however, there was a gradual increase in paclitaxel in seeds during germination (Supplementary Fig. 7).

Substantial changes in the compounds and enzymes associated with lipid metabolism were also observed in germinating seeds (Fig. 6d). There was a strong decrease in triacylglycerols (TGs), which are storage lipids in seeds, and phosphatidic acids (PAs), which are intermediates in the biosynthesis of TGs. The mRNA and protein levels of hormone-sensitive lipase (HSL) increased in germinating seeds, implying increased lipolysis of storage TGs during seed germination. Conversely, several lipids increased in germinating seeds, such as fatty acids (FAs), which are generated from TGs lipolysis, and phosphatidylcholine (PCs), which are the main structural elements of the plasma membrane. Levels of mRNA and protein for phospholipase D (PLD) declined in germinating seeds, suggesting that fewer PAs were generated from PCs, thus influencing biosynthesis. Collectively, these results indicate that the energy supply from lipids during seed germination is mainly derived from the lipolysis of TGs.

For carbohydrate metabolism, we expected glycolysis and the TCA cycle to be strongly increased in germinating seeds, considering that the energy supply of carbohydrates should be increased for embryo development. However, significant decreases were observed for several glycolysis- and TCA cycle-related enzymes (citrate synthase (CS), 1-aminocyclopropane-1-carboxylate oxidase (ACO), isocitrate dehydrogenase (IDH), succinate dehydrogenase assembly factor 2 (SDHA2), and fumarate hydratase (FUM)) and metabolites (citric acid, aconitic acid, 2-oxoglutarate, fumaric acid, and malate) in germinating seeds (Fig. 6a). In contrast, several pentose phosphate pathway-related enzymes, such as glucose-6-phosphate 1-dehydrogenase 4 (G6PD4), 6-phosphogluconolactonase 3 (6PGL3), 6-phosphogluconate dehydrogenase 3 (6PGD3), ribulose-phosphate 3-epimerase (RPE), and ribose-5-phosphate isomerase (RPI), increased in germinating seeds (Fig. 6e). Therefore, we speculated that the energy supply of carbohydrates from glycolysis and the TCA cycle was converted to pentose phosphate pathway (PPP) during Chinese yew seed germination (Fig. 6f).

### Temporal and spatial dynamics of metabolites and lipids during seed germination

**Fig. 7:**
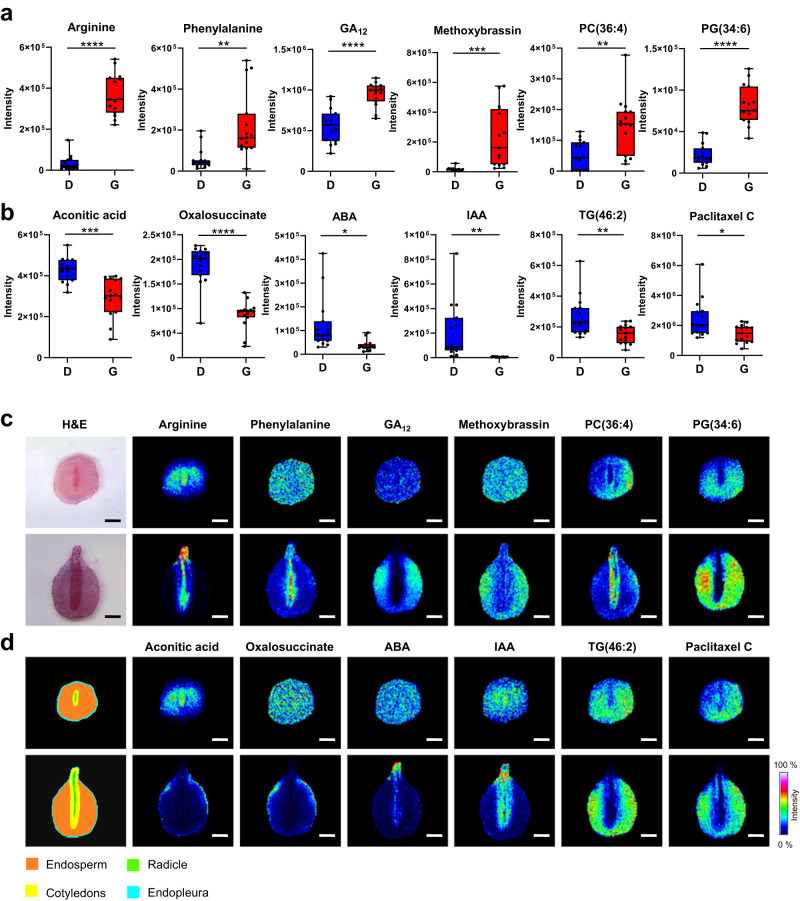
Spatial metabolome difference between dormant and germinating Chinese yew seeds. **a** Box plots of metabolites significantly increased in germinating seeds (detected by LC-MS/MS). **b** Box plots of metabolites significantly decreased in germinating seeds (detected by LC-MS/MS). The upper, middle, and lower lines correspond to the first, second, and third quartiles (25th, 50th, and 75th percentiles). Student’s *t* test, *P* value < 0.0001: ****, 0.0001 < *P* value < 0.001: ***, 0.001 < *P* value < 0.01: **, 0.01 < *P* value < 0.05: *, *P* value > 0.05: no significant. D and G represent the dormancy and germination groups, respectively. *n* = 15 biologically independent samples for each group. **c**, **d** Spatial distribution of corresponding metabolites in (**a**) and (**b**) by MALDI-MSI. The MS imaging was detected in positive-ion mode and acquired at 100 μm spatial resolution. Scale bar, 2 mm.

To determine the direct links between embryo development and metabolite function, we further visualised and compared the spatial metabolome differences of Chinese yew seeds in different physiological states using MALDI-MSI (Fig. 7 and Supplementary Data 17). The in situ visualisation results showed that L-arginine, L-phenylalanine, and PC (36:4) accumulated in germinating seeds, which was consistent with the untargeted metabolomic results (Fig. 7a, Supplementary Fig. 4e, and Supplementary Data 13). In addition, most of the upregulated amino acids and PCs were enriched in the embryos or radicles of the germinating seeds (Fig. 7c). Conversely, TCA-related compounds (aconitic acid and oxalosuccinate) and TG (46:2) were distributed in the endopleura and endosperm (Fig. 7b, d and Supplementary Data 13). For plant hormones, GA_12_ and methoxybrassin were increased in germinating Chinese yew seeds and, more precisely, were accumulated in the endosperm of germinating seeds (Fig. 7a, c). In contrast, ABA and IAA were distributed in the radicle or cotyledons of germinating seeds, further verifying the antagonistic function of ABA and GA (Fig. 7b, d). Notably, our MALDI-MSI results provided the spatial distribution information of paclitaxel C in Chinese yew seeds (mainly distributed in the endosperm) for the first time (Fig. 7d), showing a potential new perspective on the biosynthesis of the anti-tumour drug, paclitaxel. Our results suggest that seed germination (especially for embryo development) is associated with metabolite abundance as well as linked with the unambiguous spatial distribution of individual metabolite molecules.

**Fig. 8:**
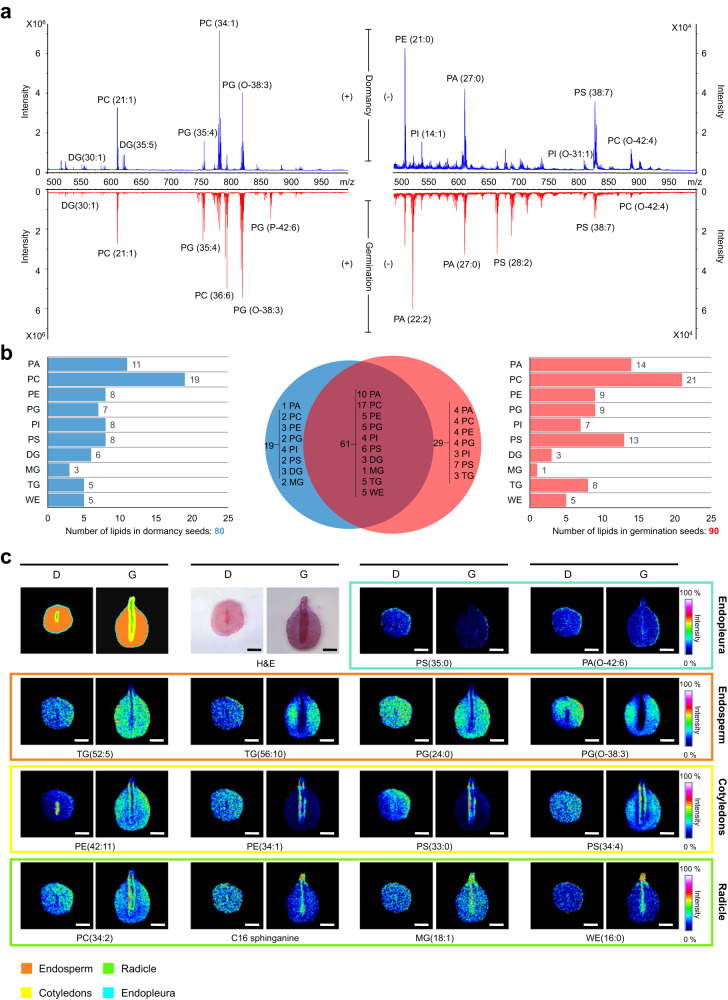
Comparison of spatial lipidome of dormant and germinating seeds by MALDI-MSI. **a** Positive ion (left) and negative ion (right) MALDI mass spectra of the dormant (up) and germinating seeds (down), respectively. **b** Venn diagram showing the classification of identified lipids from dormant (blue) and germinating (red) seeds. **c** Distinct distribution of various lipids detected from Chinese yew seeds by MALDI-MSI. D and G represent the dormancy and germination groups, respectively. Scale bar, 2 mm.

We compared the spatial lipidome differences between dormant and germinating Chinese yew seeds using the MALDI-MSI technique for the first time to further illustrate whether lipid distribution affected embryo development. Consistent with the differences detected by LC-MS/MS, eight TGs were downregulated in germinating seeds (Fig. 8a, b and Supplementary Data 17). Additionally, MALDI-MSI showed that these TGs were observed in the endosperm region (Fig. 8c), further proving that TGs are an energy source for the developing embryo until they can photosynthesise. Furthermore, 23 PCs were gathered in the radicles of germinating seeds, suggesting that the structure of the plasma membrane may change during radicle elongation (Fig. 8 and Supplementary Fig. 8). Apart from the lipids detected by LC-MS/MS and MALDI-MS/MS, distinctive spatial distribution patterns of the 86 lipids were imaged by MALDI-MSI. We found several lipids such as PS (35:1) and PA (O-42:6) was accumulated in the endopleura of dormant seeds (Fig. 8c). The distribution patterns of TGs and phosphoglycerols (PGs) were similar in germinating seeds and were observed mainly in the endosperm. In contrast, the accumulation of phosphatidylethanolamines (PEs) and phosphatidylserines (PSs) were localised in the cotyledons. Furthermore, relatively high signal intensities of sphingolipids and monoacylglycerols (MGs) were detected in the radicle of germinating seeds compared to other regions. These MALDI-MSI results revealed the temporal and spatial dynamics of lipids during seed germination.

## Discussion

The molecular mechanism of germination has been extensively studied in Arabidopsis through mutant screening and related molecular genetic studies, and many specific loci correlated with altered seed dormancy and germination have been identified, such as GA and ABA biosynthesis and signalling related genes^20^. However, it is difficult to conduct mutant screening and related molecular genetic experiments for Chinese yew seeds because it takes at least 1 year for Chinese yew seeds to germinate from deep dormancy. In this work, we found that ABA and GA_12_ contents and spatial distribution are different between dormant and germinating Chinese yew seeds. That means further detailed investigation of ABA/GA balance, plant hormone crosstalk, and especially GA (or ABA) localisation and transport is worthwhile to elucidate the molecular mechanism of Chinese yew seed germination.

Several studies have demonstrated that the ABA/GA balance (balance of hormone levels and balance of the signalling cascade) determines seed dormancy or germination^21,22^; however, the underlying molecular mechanisms are largely unknown. AP2 domain-containing transcription factors play a critical role in the dual regulation of ABA and GA biosynthesis in fine-tuning seed dormancy and germination^7^. For example, when auxin levels increase, the auxin-responsive transcription factors ARF10 and ARF16 are released to activate *ABI3* (AP2 domain-containing TF) transcription, thus maintaining seed dormancy^23,24^. In another study, CTKs exhibited an antagonistic function with ABA in seed dormancy by repressing *ABI5* transcription^25^. Other endogenous plant hormones, including JAs, SA, BRs, and SLs, can also affect the ABA/GA balance, involved in the modulation of seed dormancy and germination^26–29^. In this study, we found low endogenous ABA and high GA levels (both contents and signalling cascades) in germinating seeds, consistent with previous studies. In future studies, detailed genetic experiments and evidence of AP2-domain containing TFs are needed to fine-tune the ABA/GA balance and seed germination.

Plant hormone localisation and transport are also important for seed dormancy and germination. GA promotes seed dormancy release and germination by stimulating the expansion of the embryo and inducing hydrolytic enzymes that weaken barrier tissues such as the endosperm or testa^7^. Our multi-omics data showed that GA content, GA biosynthesis genes, and GA receptors were dramatically increased in germinating seeds, suggesting that GA has a positive correlation with seed germination consistent with several studies^20,30,31^. In addition, our MALDI-MSI results showed that GA_12_ was distributed in the endosperm of germinating Chinese yew seeds, confirming that GA could weaken the endosperm and thereby induce seed germination.

Primary and secondary metabolites play essential roles in seed germination, especially in energy supply and metabolic homoeostasis^32^. The glycolysis, tricarboxylic acid (TCA)-cycle and pentose phosphate pathway (PPP) are primarily energy metabolism pathways in plant seeds. Unlike glycolysis and the TCA cycle, PPP does not provide adenosine 5’-triphosphate (ATP) to meet the energy demands of cells. However, it supplies NADPH and ribose 5-phosphate (R5P), which are vital for the survival and proliferation of plant cells. Previous reports provide strong evidence that PPP are essential for the biosynthesis of nucleic acids and fatty acids, thereby influencing seed germination from an energy metabolism perspective^33–36^. In this work, we found higher levels of G6PD4 and 6PGD3 in germinating Chinese yew seeds than in dormant seeds, indicating that PPP-related enzymes are more active during Chinese yew seed germination. Furthermore, we found that glycolysis and TCA cycle-related metabolites and enzymes were synchronously decreased in germinating seeds, whereas the levels of PPP-related enzymes and metabolites were increased in germinating seeds. We speculate that the energy supply of carbohydrate metabolism from glycolysis and the TCA cycle was at least partially converted to PPP during Chinese yew seed germination.

We also found increased lipolysis of TGs, representing another type of primary metabolite, underpinned by decreased levels of TGs, PAs (intermediate in the biosynthesis of TGs), and TGs biosynthesis-related enzymes. Previous research has shown that TGs are the major reserves of FAs for energy production and the synthesis of carbohydrates during seed germination^37^. The storage of TGs involves primarily a universal subcellular organelle in the cytosol called the lipid droplet (LD) or oil body. During times of carbon and energy deficiency, TGs stored in LDs is hydrolysed to release FAs and other metabolites^38^. Following TGs hydrolysis by lipases, FAs are activated and transported to acyl-CoA via β-oxidation in plants^39^. Acyl-CoA is a key metabolite for energy production via mitochondrial respiration and for the synthesis of carbon skeletons via the glyoxylate cycle and gluconeogenesis. Therefore, we believe that the energy supply from lipids during Chinese yew seed germination is mainly derived from the lipolysis of TGs.

In addition, spatially heterogeneous localisation of 117 lipids was noticeably observed across the entire seed tissue section using MALDI-MSI. Specifically, PCs and sphingolipids tend to be enriched in the embryos and radicles of germinating seeds, whereas TGs and PGs are more enriched in the endosperm of dormant seeds. Plant sphingolipids perform critical functions, such as structural roles in maintaining membrane integrity, which can be achieved by their clustering with sterols forming highly dynamic microdomains called lipid rafts located in the plasma membrane^40,41^. Most sphingolipid profiles were enhanced in embryos of germinating seeds, implying that more signal transduction events occurred in this region. Furthermore, the distribution of membrane lipids in Chinese yew seeds was asymmetric, with PCs in the embryo, for example, whereas PGs were primarily in the endosperm. Such local phospholipid distribution imbalances soften the tissue structure around the embryo, foster embryo development, and facilitate radicle elongation.

Together, we have generated a comprehensive multi-omics approach to investigate the germination mechanisms of Chinese yew seeds. This approach sheds light on the transcriptome, proteome, metabolome, and spatial lipidome of Chinese yew seeds, as well as helps understand the regulation of seed germination by gene transcription, protein translation, and metabolic homoeostasis, especially by plant hormone regulation. In addition, investigating different levels of omics data, for example, Chinese yew seeds, can lead to new insights into biological processes. Therefore, we anticipate that this analytical strategy, along with spatial-omics, will enable researchers to perform many types of systems-level analyses that are not covered in this study. We also expect this study to affect plant research more broadly, as the study shows that state-of-the-art mass spectrometry-based assays can achieve qualitative and quantitative plant research with a unique spatial distribution of molecules.

## Methods

### Plant materials and growth conditions

Chinese yew seeds with uniform sizes were collected using a five-point sampling method from Dahetang Village of Ruichang City, Jiangxi Province (N 115°23.14, S 29°55.07, 700 m) in November 2017. Seeds were stored in wet sand (25 ± 0.5 °C) for 30 days to eliminate the raw material differences. After acclimation, seeds were collected and frozen at −80 °C for further analysis. Seeds prepared for germinating were treated with 98% H_2_SO_4_ for 1 min, followed by rinsing under clean running water for 24 h and then 330 days wet-sand-stratification (90 days at 4 °C, 90 days at 25 °C, and 150 days at 4 °C) for the germination.

### Reagents and standards

2-Mercaptobenzothiazole (2-MBT), α-cyano-4-hydroxycinnamic acid (CHCA), LC/MS-grade acetonitrile (ACN), methanol, ethanol, trifluoroacetic acid (TFA), formic acid (FA), haematoxylin and eosin (H&E) staining solutions were purchased from Sigma-Aldrich (St. Louis, MO). Standard compound of arginine, phenylalanine, GA_12_, methoxybrassin, aconitic acid, oxalosuccinate, ABA, IAA, paclitaxel C, PC (36:4), PG (34:6), and TG (46:2) were purchased from Sigma-Aldrich (St. Louis, MO). Ultrapure water used throughout the experiment was obtained from a Milli-Q system (Millipore, Milford, MA).

### RT-qPCR analysis

Total RNA was isolated by using EasyPure Plant RNA kit (TIANGEN), then converted to complementary DNA (cDNA) using the TransScript One-Step gDNA Removal and cDNA Synthesis SuperMix (TRANSGENE BIOTECH CO., LTD.). qRT-PCR was performed with a 20 μL reaction mixture containing 2 μL of cDNA template with Perfect Start Green qPCR SuperMix (TRANSGENE BIOTECH CO., LTD.) on the CFX96™ Real-Time PCR Detection System (Bio-Rad, USA). Mean cycle threshold (Ct) values were normalised using ACTIN^17^ and GPADH^42^ reference gene as internal control. The relative transcript abundance for each gene was determined by 2^−^^ΔΔCt^ method. Three technical replicates and three biological replicates were analysed for each mRNA. Primer sequences of each mRNA are listed in Supplementary Data 18.

### RNA sequencing, transcriptome de novo assembly, annotation, and expression

Total RNA was isolated and enriched using the Dynabeads mRNA Purification Kit (Merck), according to the manufacturer’s instructions. Briefly, 80 mg of seed samples (powder ground with liquid nitrogen) was used to extract total RNA using the CTAB-PBIOZOL reagent. After ethanol precipitation, the RNA pellet was washed with 75% ethanol, and the final dried pellet was resuspended in DEPC-treated water for subsequent analysis. Three independent biological replicates were performed.

Raw RNA-seq reads were filtered to remove reads containing adaptors, poly-N, or low-quality reads using SOAPnuke (v.1.4.0). After filtering, clean reads were aligned with reference gene sequences by Bowtie2 (v.2.2.5). Clean reads were de novo assembled using Trinity software (v.2.0.6), and redundancy was eliminated using Tgicl (v.2.0.6) to obtain unigenes. TransDecoder (v.3.0.1) was used to predict candidate coding regions within the transcript sequences. All assembled unigenes were searched against seven databases using BLAST (v.2.2.23), including non-redundant (NR) protein, nucleotide (NT), Swiss-Prot, clusters of eukaryotic Orthologous Groups (KOG), Gene Ontology (GO), Kyoto Encyclopedia of Genes and Genomes (KEGG), and Pfam. Transcription factors (TFs) were predicted by extracting open reading frames (ORF) of unigenes (getorf, v.6.5.7.0) and searching for transcription factor protein domains using HMMER (v.3.0). Transcript abundances were quantified using the RSEM software tool (v.12.8), and mRNA quantities are displayed as fragments per kilobase of transcript per million mapped reads (FPKM). Differentially expressed genes (DEGs) were identified using DEseq2 (Q value <=0.05, fold change >=2). GO enrichment analysis was performed using Phyper (function from R). The significance levels of GO terms were corrected using the Q value with a rigorous threshold (Q value < 0.05).

### SDS-PAGE

In all, 20 µg of protein for each sample were mixed with 5× loading buffer respectively and boiled for 5 min. The proteins were separated on 12.5% SDS-PAGE gel (constant current 14 mA, 90 min). Protein bands were visualised by Coomassie Blue R-250 staining.

### Proteome profiling

Frozen Chinese yew seeds were ground in liquid nitrogen inside a mortar and using a pestle. Proteins were extracted using a SDT buffer (4% SDS, 1 mM DTT, and 100 mM Tris-HCl, pH 7.6). The concentration of each sample was quantified using the BCA Protein Assay Kit (Bio-Rad, USA). Proteins were precipitated overnight with 10% trichloroacetic acid in acetone at −20 °C and subsequently washed twice with ice-cold acetone. Dry samples were incubated with urea digestion buffer (8 M urea, 50 mM Tris-HCl pH 7.5, 1 mM DTT, complete EDTA-free protease inhibitor cocktail (PIC) (Roche), phosphatase inhibitor (PI-III; in-house, composition resembling phosphatase inhibitor cocktail 1, 2, and 3 from Sigma-Aldrich)) for 1 h. Protein concentration was determined with a Bradford assay. Then, 1 mg of protein was reduced (10 mM dithiothreitol (DTT), 1 h, room temperature), alkylated (55 mM chloroacetamide, 30 min, room temperature) and subsequently diluted 1:8 with digestion buffer (50 mM Tris-HCl pH 8.0, 1 mM CaCl_2_). In-solution pre-digestion with trypsin (Roche) was performed for 4 h at 37 °C (1:100 protease:protein ratio), followed by overnight digestion with trypsin (1:100 protease:protein ratio). Samples were acidified to pH 3 using trifluoroacetic acid (TFA) and centrifuged at 14,000 × *g* for 15 min at 4 °C. The supernatant was desalted on 50 mg SepPAC SPE catridges (Waters). Peptides were eluted with 0.1% TFA in 50% acetonitrile (ACN) and vacuum-dried down in a Thermo Savant SPD SpeedVac (Thermo Fisher Scientific).

The tryptic peptides of each sample were desalted on a C18 cartridge (Empore™ SPE Cartridges C18, bed I.D. 7 mm, volume 3 mL, Sigma), concentrated by speed-vacuum centrifugation, and reconstituted in 40 µL of 0.1% (v/v) formic acid. An amount of the peptide mixture, equivalent to 100 µg of protein for each sample, was labelled using 6-TMT reagent according to the manufacturer’s instructions (Thermo Scientific). Strong cation exchange (SCX) chromatography was performed for fractionation of tryptic peptides on an AKTA Purifier system (GE Healthcare). Finally, the collected fractions were acidified with formic acid, desalted on a C18 cartridge, dried and then reconstituted in 0.1% formic acid for the following liquid chromatography–tandem mass spectrometry (LC–MS/MS) analysis. Three biological replicates were performed for each assay.

Nanoflow LC–MS/MS was performed by coupling an Easy nLC (Proxeon Biosystems, now Thermo Fisher Scientific) to a QExactive Orbitrap HF mass spectrometer (Thermo Scientific). Peptide loading and washing were performed on a trap column (Thermo Scientific Acclaim PepMap100, 100 μm × 2 cm, nanoViper C18) at a flow rate of 5 μL/min with the 100% loading buffer (0.1% formic acid) for 10 min. Peptide separation was performed on an analytical column (Thermo Scientific Easy C18-A2, 10 cm, 75 μm inner diameter, 3 μm particle size) at a flow rate of 300 nL/min using a 90 min gradient of 4% to 32% solvent B (solvent A: 0.1% formic acid in water; solvent B: 0.1% formic acid in 84% aqueous acetonitrile). The mass spectrometer was operated in the positive ion mode. LC-MS/MS data were acquired using a data-dependent Top10 method, dynamically choosing the most abundant precursor ions from the survey scan (300–1800 *m*/*z*) for HCD fragmentation. The automatic gain control (AGC) target was set to 3E6, and the maximum injection time was set to 10 ms. The dynamic exclusion duration was 40 s. Survey scans were acquired at a mass resolution of 70,000 full width at half-maximum (FWHM) at *m*/*z* 200, the mass resolution for HCD spectral acquisition was set to 17,500 at *m*/*z* 200, and the isolation width was 2 Da. The normalised collision energy was 30 eV, and the underfill ratio, which specified the minimum percentage of the target value likely to be reached at the maximum fill time, was defined as 0.1%. The instrument was run using the peptide recognition mode.

The acquired MS/MS raw data from each sample were searched using the MASCOT engine (Matrix Science, London, UK; version 2.2) embedded into Proteome Discoverer 1.4 software for identification analysis. Protein abundance estimation was based on intensity-based absolute quantification (TMT). The subcellular localisation of the differentially expressed proteins was predicted using CELLO. Subsequently, differentially expressed proteins were blasted against the KEGG database to retrieve their KEGG orthology identification. Enrichment analysis was applied based on Fisher’s exact test, considering all quantified proteins as the background dataset. The Benjamini–Hochberg correction for multiple testing was further applied to adjust the derived *P* values. Only functional categories and pathways with *P* values under a threshold of 0.05 were considered significant.

### MS-based untargeted metabolomics

Frozen Chinese yew seeds were ground to fine powder in liquid nitrogen. 100 mg of the powder of each sample was weighed into a 2-mL pre-cooled Eppendorf tube. Ice-cold extraction solvents were added to the tube and the powder was homogenised with the aid of 5-mm stainless steel beads on a Precellys tissue homogeniser (Bertin Technologies, France). Twelve combinations of extraction solvents, including methanol/water (95:5, 80:20, 50:50, v/v), acetonitrile/water (95:5, 80:20, 50:50, v/v), ethyl acetate/water (95:5, 80:20, 50:50, v/v), and methanol/chloroform/water (90:5:5, 80:10:10, 60:20:20, v/v/v), were prepared and tested for extractions in order to select the optimal extraction solvent. After homogenisation on the tissue homogeniser for 60 s at a vibrating frequency of 30 Hz, the samples were centrifuged for 10 min at 4 °C and 13,500 × *g* in an Eppendorf 5430 R centrifuge. The supernatants were carefully collected and filtered through a 0.22 μm PTFE filter membrane before LC-MS/MS analysis. Fifteen biological replicates were performed for LC-MS/MS.

A Dionex UltiMate 3000 ultrahigh-performance liquid chromatography (UHPLC) system (Thermo Fisher Scientific, MA, USA) coupled to a Bruker Impact II quadrupole time-of-flight (QTOF) mass spectrometer (Bruker Daltonics, Billerica, MA, USA) was used to acquire the metabolomic data. A Waters ACQUITY UPLC BEH C18 column (1.7 μm, 2.1 × 50 mm) was used to separate metabolites in the extracts with a mobile phase consisting of solvent A (water containing 0.1% formic acid) and solvent B (acetonitrile containing 0.1% formic acid) at a flow rate of 0.3 mL/min. The gradient elution conditions were set as follows:0–5 min, 5–35% B; 5–20 min, 35–65% B; 20–25 min, 65–100% solvent B; 25–35 min, 100% solvent B; 35–35.1 min, 100–5% solvent B and equilibration at 5% B for 5 min. The injection volume for each sample was 10 μL. Mass spectrometry detection was performed both in positive and negative ion mode with full-mass detection for relative quantitation. LC-MS/MS was carried out in an automatic way. The ESI capillary and end-plate offset voltages were set to 4500 and 500 V, respectively. High-purity nitrogen was used as the dry gas at a flow rate of 8 L/min for the desolvation and desorption of droplets. The dry temperature was 220 °C. For detection, LC-MS data were acquired in the mass range of *m*/*z* 50–1300 Da. For the MS/MS scans, the three most abundant ions on each full-mass survey scan were selected as precursor ions for subsequent fragmentation with 30% normalised collision energy using collision-induced dissociation (CID).

Raw data were converted to mzXML files using the ProteoWizard MSConvertGUI program (Palo Alto, CA, USA). Data processing, including feature detection, retention time shift correction, peak picking, peak alignment and feature annotation, was performed using XCMS Online (https://xcmsonline.scripps.edu/) with the embedded R packages including *xcms*, *biobase*, *multtest*, and *CAMERA*. LC-MS/MS data was acquired in both positive and negative ion mode, and data processing was achieved by *xcms* package based on R language. Centwave method was used for feature detection, and obiwarp method was used for retention time correction. For peak alignment, bandwidth (standard deviation or half width at half maximum) was set as 5, and width of overlapping m/z slices was set as 0.015 for creating peak density chromatograms and grouping peaks across samples. The detailed parameter settings for LC-MS/MS dataset processing are listed in Supplementary Table 1.

Statistical analyse such as *t* tests, fold change analysis, correlation analysis, significance analysis of metabolites (SAM), and empirical Bayesian analysis of metabolites (EBAM) was performed using MetaboAnalyst 5.0 (https://www.metaboanalyst.ca/). Principal component analysis (PCA) and orthogonal partial least squares-discriminant analysis (OPLS-DA) were performed using the *SIMCA-P* 15.0 (Umetrics, Umea, Sweden) software. The MS and MS/MS spectra of the differentially expressed metabolites were searched against the METLIN, LIPID MAPS, and MASSBANK databases for matching the possible metabolite candidates. Commercially available reference compounds were purchased and used to confirm the identities including differentiation of metabolite isomers. Differentially expressed metabolites are shown as clouds plotted based on XCMS Online. Enrichment and functional analyses of the differentially expressed metabolites were performed using MetaboAnalyst 5.0.

### Targeted detection of GA_3_, GA_4_, and GA_7_ by UHPLC-MS/MS

The extraction of the targeted metabolites was similar to the untargeted metabolomics experiment except for the extraction solvent and the gradient elution conditions of the UHPLC. Methanol/water (80:20, v/v) was prepared for the extraction of GA_3_, GA_4_, and GA_7_. A Waters ACQUITY UPLC BEH C18 column (1.7 μm, 2.1 × 50 mm) was used to separate metabolites in the extracts with a mobile phase consisting of solvent A (water containing 0.1% formic acid) and solvent B (acetonitrile containing 0.1% formic acid) at a flow rate of 0.2 mL/min. The gradient elution conditions were set as follows: 0–20 min, 5–100% solvent B; 20–30 min, 100% solvent B; 30–30.1 min, 100–5% solvent B and equilibration at 5% B for 10 min. The injection volume for each sample was 20 μL. Three biological replicates of dormant and germinating Chinese yew seeds were used for targeted metabolites detection.

### Joint analysis of the transcriptome, proteome, and metabolome data

The scatter plots of median transcript (FPKM) and protein abundance (TMT) in dormant and germinating seeds are displayed with their marginal histograms. Genes detected at protein and transcript levels were assigned to the core datasets (*n* = 5496). The core dataset was used for all further calculations involving the protein-to-transcript (PTR) ratio. PTR values were calculated by calculating the ratio between protein abundance and the corresponding transcript abundance for each gene. The PTR value distribution was plotted for the dormant and germinating seeds. Variations in PTR values indicate whether protein and transcript levels of a given gene are regulated in similar (stable PTR, fold change <1.5) or different ways (fluctuating PTR) between dormant and germinating seeds. The percentage of genes with stable or fluctuating PTR was calculated and displayed as an arrow.

The protein-metabolite interaction network was explored and visualised using MetaboAnalyst 5.0 (https://www.metaboanalyst.ca/). Genes with fluctuating PTR and differentially expressed metabolites were superimposed onto metabolic pathway maps based on the KEGG pathway. In addition, differentially expressed transcripts, proteins, and metabolites involved in synthesis and metabolism of phytohormones, amino acids, terpenoids (e.g., paclitaxel), alkaloid biosynthesis, lipid metabolism and carbohydrate metabolism were also depicted based on the KEGG pathway.

### MALDI-MSI based spatial metabolome and lipidome

Chinese yew seeds were sectioned at −18 °C using a Leica CM1860 cryostat (Leica Microsystems Inc., Wetzlar, Germany). Serial tissue slices of 20-μm thickness were transferred onto indium tin oxide (ITO)-coated microscopic glass slides (Bruker Daltonics, Billerica, MA) by thaw-mounting and stored at −80 °C. Optical images of the tissue sections were captured using a flatbed scanner (Epson Perfection V550; Suwa, Japan). The sections were desiccated at room temperature for 15 min before the application of MALDI matrix. The matrix solution was prepared by dissolving 2-mercaptobenzothiazole (2-MBT) in a mixed methanol/water/formic acid (80:18:2, v/v/v) solution at a concentration of 10 mg/mL. For matrix coating, the 2-MBT solution was applied to the surfaces of the sliced seed tissue sections using a GET-Sprayer (І) (HIT Co., Ltd., Beijing, China). Approximately 40 cycles (2 s spray, 30 s incubation, and 60 s drying time) were performed for the matrix coating. After MALDI-MSI experiments, histological staining of the tissue sections was performed using H&E.

MALDI-MS measurements were performed both in the positive-ion and negative-ion reflection mode on an Autoflex Speed MALDI-TOF/TOF mass spectrometer (Bruker Daltonics, Billerica, MA) equipped with a Smartbeam II 2 kHz laser. For profiling of metabolites and lipids, all mass spectra were acquired over a mass range of 100–1500 Da and were recorded by accumulating 14 scans at 500 laser shots per scan. The mass spectra was acquired by applying an optimised laser power for 2-MBT at 50% of the full scale of the Nd:YAG UV laser power with a global attenuator offset of 20%, which is ca. 1.6 mJ per pulse, according to the manufacturer’s product specification (corresponding to 800 μJ per laser shot). MS/MS spectra were acquired in CID mode and argon was used as the collision gas. The metabolite fragment ions were acquired under the following parameters: ion source I, 19.0 kV; ion source II, 17.4 kV; lens, 8.8 kV; reflector I, 21.0 kV; reflector II, 9.8 kV; and accelerating voltage, 20.0 kV. The UV laser power ranged from 65% to 90%. MS/MS spectra were recorded from based on no less than 5000 laser shots over the *m*/*z* range of 0 to 100 with a sampling rate of 2.00 G/s, a detector gain of 9.5×, and an electronic gain of 100 mV. Six replicates per tissue section were performed for profiling. For MALDI MS imaging, the ion images of endogenous metabolites in seeds were obtained at a spatial resolution of 100 μm, with 500 laser shots per ablated spot.

For the MS profiling data, the ion signals of a mixed standard solution of bradykinin 1–7 ([M + H]^+^, *m*/*z* 757.40), angiotensin II ([M + H]^+^, *m*/*z* 1046.54), angiotensin I ([M + H]^+^, *m*/*z* 1296.68), and substance P ([M + H]^+^, *m*/*z* 1347.74), combined with the matrix ions of CHCA ([M-H_2_O + H]^+^, *m*/*z* 172.04; [M + H]^+^, *m*/*z* 190.05; [M+Na]^+^, *m*/*z* 212.03; [2 M + H]^+^, *m*/*z* 379.09; [3 M + H]^+^, *m*/*z* 568.16), were used for external mass calibration using the cubic-enhanced mode. *FlexAnalysis* software (Bruker Daltonics, v.3.4) was used for viewing and processing of the profiling data, including peak alignment, monoisotopic “peak picking”, and signal-to-noise threshold setting.

Ion images were reconstructed and visualised using *FlexImaging* software (Bruker Daltonics, v.4.1) with an allowable mass filter width of 10 ppm. Ion images of the detected metabolites and lipids were exported using the interpolated pixel display option.

### Histological staining

After MALDI-MSI experiments, the tissue sections were washed with 70, 90, and 100% methanol solutions in sequence to remove the matrix, and then H&E staining was performed to obtain standard histological optical images. Tissue sections of *T. mairei* seed were successively immersed into 95% and 70% ethanol for 30 s, followed by 2-min haematoxylin solution staining. Next, these tissue sections were immersed into 70% and 95% ethanol for 30 s. After staining in eosin solution for 1 min, tissue sections were finally washed by 95% and 100% ethanol for 30 s.

### Statistics and reproducibility

The experiments of transcriptome and proteome were performed in triplicates and data were averaged from three independent experiments and presented as mean ± SD. Fifteen biological replicates were performed for LC-MS/MS. Student’s *t* test for comparisons was used to perform statistical analysis. Significance was determined at *P* value as indicated in the figure legends. NS: no significance at *P* > 0.05; *P < 0.05, **P < 0.01, ***P < 0.001, and ****P < 0.0001: statistically significant at the level as indicated.

### Reporting summary

Further information on research design is available in the Nature Portfolio Reporting Summary linked to this article.

### Supplementary information

Supplementary information

Description of Additional Supplementary Files

Supplementary Data 1

Supplementary Data 2-7

Supplementary Data 8-12

Supplementary Data 13-18

Reporting Summary

## Data Availability

Source data for Fig. 1i can be found in Supplementary Data 1, 9, and 15. Source data for Fig. 2a–e can be found in Supplementary Data 8. Source data for Fig. 2f, g can be found in Supplementary Data 10. Source data for Fig. 2h can be found in Supplementary Data 11. Source data for Fig. 2i, j can be found in Supplementary Data 7. Source data for Fig. 3d–e, h can be found in Supplementary Data 12. Source data for Fig. 4d–g can be found in Supplementary Data 14. Source data of Fig. 7a, b can be found in Supplementary Data 13. Source data of Fig. 8a can be found in Supplementary Data 17. The RNA-seq data has been deposited at NCBI Sequence Read Archive database under BioProject no. PRJNA980442. The mass spectrometry proteomics data have been deposited to the ProteomeXchange Consortium (http://proteomecentral.proteomexchange.org) via the iProX partner repository with the dataset identifier PXD042641.

## References

[1] Wani MC, Taylor HL, Wall ME, Coggon P, McPhail AT (1971). Plant antitumor agents. VI. The isolation and structure of taxol, a novel antileukemic and antitumor agent from *Taxus brevifolia*. J. Am. Chem. Soc..

[2] Zhang JT, Ru W (2010). Population characteristics of endangered species *Taxus chinensis* var. *mairei* and its conservation strategy in Shanxi, China. Popul. Ecol..

[3] Li N (2014). Avian seed dispersal and seedling distribution of the endangered tree species, *Taxus chinensis*, in patchy habitats. Plant Ecol. Divers.

[4] Li C, Huo C, Zhang M, Shi Q (2008). Chemistry of Chinese yew, *Taxus chinensis* var. *mairei*. Biochem. Syst. Ecol..

[5] Baskin JM, Baskin CC (2007). A classification system for seed dormancy. Seed Sci. Res..

[6] Liu D, Yu HL, Li FL, Guo HH (2011). An analysis of dormancy and dormancy release in *Taxus chinensis* var. *mairei* seeds. Seed Sci. Technol..

[7] Shu K, Liu XD, Xie Q, He ZH (2016). Two faces of one seed: hormonal regulation of dormancy and germination. Mol. Plant.

[8] Li Z, Sheerin DJ, von Roepenack-Lahaye E, Stahl M, Hiltbrunner A (2022). The phytochrome interacting proteins ERF55 and ERF58 repress light-induced seed germination in *Arabidopsis thaliana*. Nat. Commun..

[9] Li H (2021). Melatonin antagonizes ABA action to promote seed germination by regulating Ca^2+^ efflux and H_2_O_2_ accumulation. Plant Sci..

[10] Dorone Y (2021). A prion-like protein regulator of seed germination undergoes hydration-dependent phase separation. Cell.

[11] Liu Y, Koornneef M, Soppe WJ (2007). The absence of histone H2B monoubiquitination in the *Arabidopsis hub1* (*rdo4*) mutant reveals a role for chromatin remodeling in seed dormancy. Plant Cell.

[12] Tognacca RS, Botto JF (2021). Post-transcriptional regulation of seed dormancy and germination: Current understanding and future directions. Plant Commun..

[13] Bunsick M (2020). *SMAX1*-dependent seed germination bypasses GA signalling in *Arabidopsis* and *Striga*. Nat. Plants.

[14] Wang Z (2016). *Arabidopsis* seed germination speed is controlled by SNL histone deacetylase-binding factor-mediated regulation of *AUX1*. Nat. Commun..

[15] Wang Y (2020). Abscisic acid promotes jasmonic acid biosynthesis via a ‘SAPK10-bZIP72-*AOC*’ pathway to synergistically inhibit seed germination in rice (*Oryza sativa*). N. Phytol..

[16] Ju L (2019). JAZ proteins modulate seed germination through interaction with ABI5 in bread wheat and *Arabidopsis*. N. Phytol..

[17] Xiong X (2021). The *Taxus* genome provides insights into paclitaxel biosynthesis. Nat. Plants.

[18] Zhang Y, Scossa F, Fernie AR (2021). The genomes of *Taxus* species unveil novel candidates in the biosynthesis of taxoids. Mol. Plant.

[19] Mergner J (2020). Mass-spectrometry-based draft of the *Arabidopsis* proteome. Nature.

[20] Holdsworth MJ, Bentsink L, Soppe WJJ (2008). Molecular networks regulating *Arabidopsis* seed maturation, after-ripening, dormancy and germination. N. Phytol..

[21] Seo M (2006). Regulation of hormone metabolism in *Arabidopsis* seeds: phytochrome regulation of abscisic acid metabolism and abscisic acid regulation of gibberellin metabolism. Plant J..

[22] Shu K (2013). ABI4 regulates primary seed dormancy by regulating the biogenesis of abscisic acid and gibberellins in *Arabidopsis*. PLoS Genet..

[23] Ding ZJ (2014). WRKY41 controls Arabidopsis seed dormancy via direct regulation of *ABI3* transcript levels not downstream of ABA. Plant J..

[24] Liu X (2013). Auxin controls seed dormancy through stimulation of abscisic acid signaling by inducing ARF-mediated *ABI3* activation in *Arabidopsis*. Proc. Natl Acad. Sci. USA.

[25] Wang Y (2011). Cytokinin antagonizes ABA suppression to seed germination of *Arabidopsis* by downregulating ABI5 expression. Plant J..

[26] Jacobsen JV (2013). Roles for blue light, jasmonate and nitric oxide in the regulation of dormancy and germination in wheat grain (*Triticum aestivum* L.). Planta.

[27] Xie Z, Zhang ZL, Hanzlik S, Cook E, Shen QJ (2007). Salicylic acid inhibits gibberellin-induced alpha-amylase expression and seed germination via a pathway involving an abscisic-acid-inducible *WRKY* gene. Plant Mol. Biol..

[28] Xi W, Liu C, Hou X, Yu H (2010). *MOTHER OF FT AND TFL1* regulates seed germination through a negative feedback loop modulating ABA signaling in *Arabidopsis*. Plant Cell.

[29] Toh S (2012). Thermoinhibition uncovers a role for strigolactones in *Arabidopsis* seed germination. Plant Cell Physiol..

[30] Yamauchi Y (2007). Contribution of gibberellin deactivation by AtGA2ox2 to the suppression of germination of dark-imbibed *Arabidopsis thaliana* seeds. Plant Cell Physiol..

[31] Hu Y (2018). Gibberellins play an essential role in late embryogenesis of *Arabidopsis*. Nat. Plants.

[32] Sechet J (2016). Xyloglucan metabolism differentially impacts the cell wall characteristics of the endosperm and embryo during *Arabidopsis* seed germination. Plant Physiol..

[33] Swamy PM, Sandhyarani CK (1986). Contribution of the pentose phosphate pathway and glycolytic pathway to dormancy breakage and germination of peanut (*Arachis hypogaea* L.) Seeds. J. Exp. Bot..

[34] Zhu M (2023). Insights into the regulation of energy metabolism during the seed-to-seedling transition in marine angiosperm *Zostera marina* L.: Integrated metabolomic and transcriptomic analysis. Front. Plant Sci..

[35] Andriotis VME, Smith AM (2019). The plastidial pentose phosphate pathway is essential for postglobular embryo development in Arabidopsis. Proc. Natl Acad. Sci. USA.

[36] Nwafor CC (2022). Genetic and Biochemical investigation of seed fatty acid accumulation in *Arabidopsis*. Front Plant Sci..

[37] Graham IA (2008). Seed storage oil mobilization. Annu. Rev. Plant Biol..

[38] Xu C, Shanklin J (2016). Triacylglycerol metabolism, function, and accumulation in plant vegetative tissues. Annu. Rev. Plant Biol..

[39] Theodoulou FL, Eastmond PJ (2012). Seed storage oil catabolism: a story of give and take. Curr. Opin. Plant Biol..

[40] Lingwood D, Simons K (2010). Lipid rafts as a membrane-organizing principle. Science.

[41] Mongrand S, Stanislas T, Bayer EM, Lherminier J, Simon-Plas F (2010). Membrane rafts in plant cells. Trends Plant Sci..

[42] Chen Y (2021). Salicylic acid-responsive factor TcWRKY33 positively regulates taxol biosynthesis in *Taxus chinensis* in direct and indirect ways. Front. Plant Sci..

