# Supplementary material for: Insights from multi-omics integration into seed germination of Taxus chinensis var mairei
Source: Commun Biol. 2023 Sep 11;6:931. doi: 10.1038/s42003-023-05307-x (PMC10495361; doi:10.1038/s42003-023-05307-x)
Supplement: Supplementary file 1 — Supplementary information [file 42003_2023_5307_MOESM1_ESM.pdf]

Supplementary information

Mass spectrometry-based multi-omics insights into seed germination of *Taxus chinensis* var *mairei*

Lulu Chen[1,2#], Liang Qin[1,3#], Yawen Zhang[1,3#], Hualei Xu[1,3], Yufen Bu[2,4], Ran Wu[1,3], Haiqiang Liu[1,3], Qichen Hao[1,3], Hao Hu[1,3], Yijun Zhou[1,3], Jinchao Feng[1,3], Yanping Jing[2,4*], Jun Han[5,6*], and Xiaodong Wang[1,3*]

1. College of Life and Environmental Sciences, Centre for Imaging & Systems Biology, Minzu University of China, Beijing 100081, China.
2. National Engineering Research Center of Tree Breeding and Ecological Restoration, College of Biological Sciences and Biotechnology, Beijing Forestry University, Beijing, 100083, China.
3. Key Laboratory of Mass Spectrometry Imaging and Metabolomics (Minzu University of China), State Ethnic Affairs Commission, Beijing 100081, China.
4. Key Laboratory of Genetics and Breeding in Forest Trees and Ornamental Plants, Ministry of Education, College of Biological Sciences and Biotechnology, Beijing Forestry University, Beijing, 100083, China.
5. Genome British Columbia Proteomics Centre, University of Victoria, Victoria, BC V8Z 7X8, Canada.
6. Division of Medical Sciences, University of Victoria, Victoria, BC V8P 5C2, Canada.

#These authors contributed equally to this work.

***Corresponding authors：**

Prof. Xiaodong Wang, Ph.D

#27 Zhongguancun South Avenue, Beijing 100081, China.

Email: Xiaodong@muc.edu.cn; **Tel.:** +86-10-68932922; **Fax:** +86-10-68936927

Dr. Jun Han

#3101-4464 Markham St., Vancouver Island Technology Park, Victoria, BC V8Z 7X8, Canada.

Email: hanjun@uvic.ca; **Tel.:** (250) 483-3235

Prof. Yanping Jing, Ph.D

#35 Qinghua East Road, Beijing, 100083, China.

Email: ypjing@bjfu.edu.cn; **Tel.:** +86-10-62336164; **Fax:** +86-10-62336013

This PDF file includes:

1. Supplementary Table 1

2. Supplementary Figures 1-8

Supplementary Table 1. Parameter of feature detection method (centWave) for LC-MS/MS dataset processing.

Setting	Note	Value
ppm	parts per million	10
minimum peak width	minimum chromatographic peak width in seconds	5
maximum peak width	maximum chromatographic peak width in seconds	20
mzdiff	minimum difference in m/z for peaks with overlapping retention times	0.01
S/N	signal/noise threshold	6
prefilter peaks	minimum peaks of mass traces that can be retained	3
prefilter intensity	minimum peak intensity of mass traces that can be retained	100

1. Supplementary Figures

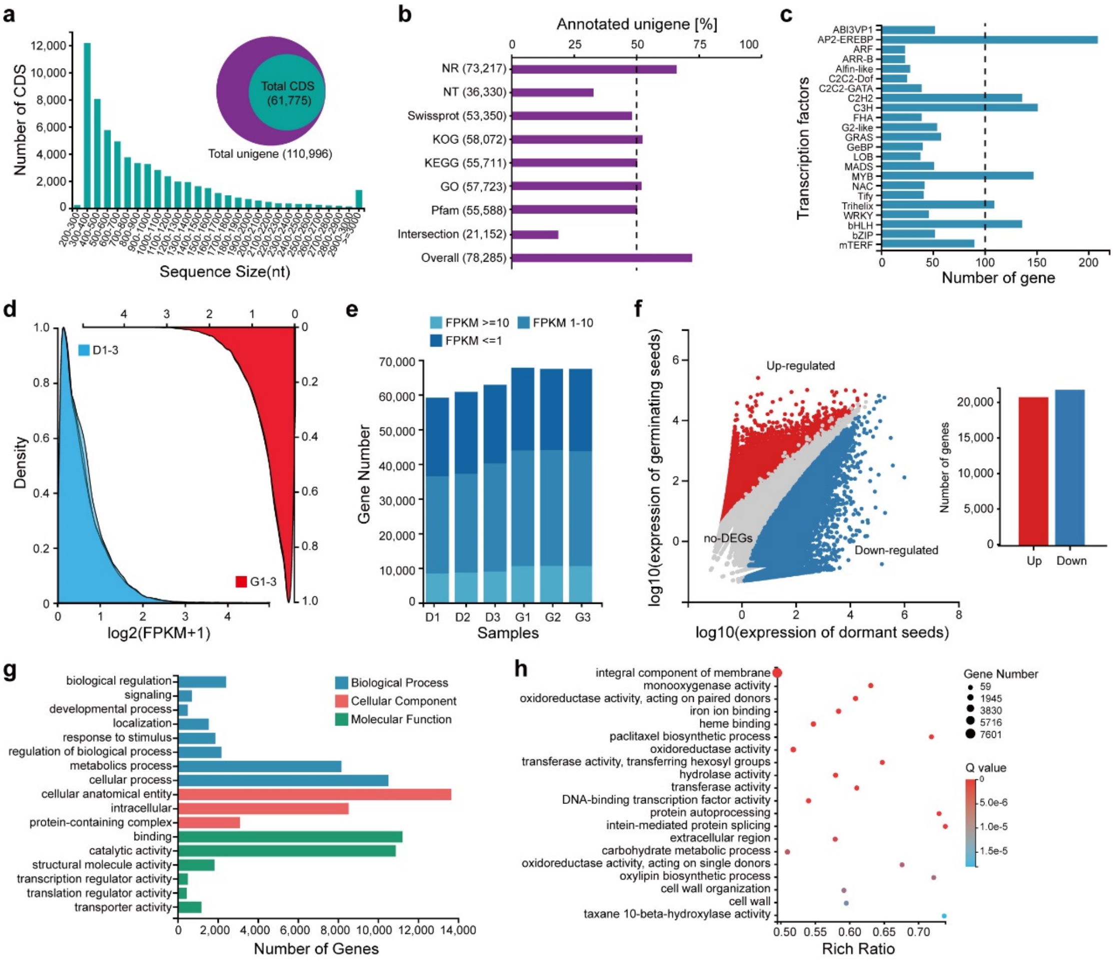

Supplementary Fig. 1. Descriptive analysis of the transcriptome of Chinese yew seeds. a, The inset shows the total number of non-redundant unigenes and the overlap of the predicted CDS. The length distribution of CDS is shown in the histogram. **b**, Percentage of unigenes annotated using seven databases. **c**, Number of genes annotated to transcription factors family. **d**, Dynamic range of transcript abundance in dormant and germinating seeds. **e**, Number of transcripts distributed in different partitions of dormant and germinating seeds (Three replicates were performed of dormant and germinating seeds). **f**, Left, scatter plot showing differentially expressed transcripts between dormant and germinating seeds. Right, the number of upregulated and downregulated transcripts (G vs D). **g**, GO terms of unigenes were assigned to biological, metabolic, and cellular processes. **h**, GO enrichment analysis of differentially expressed transcripts between dormant and germinating seeds.

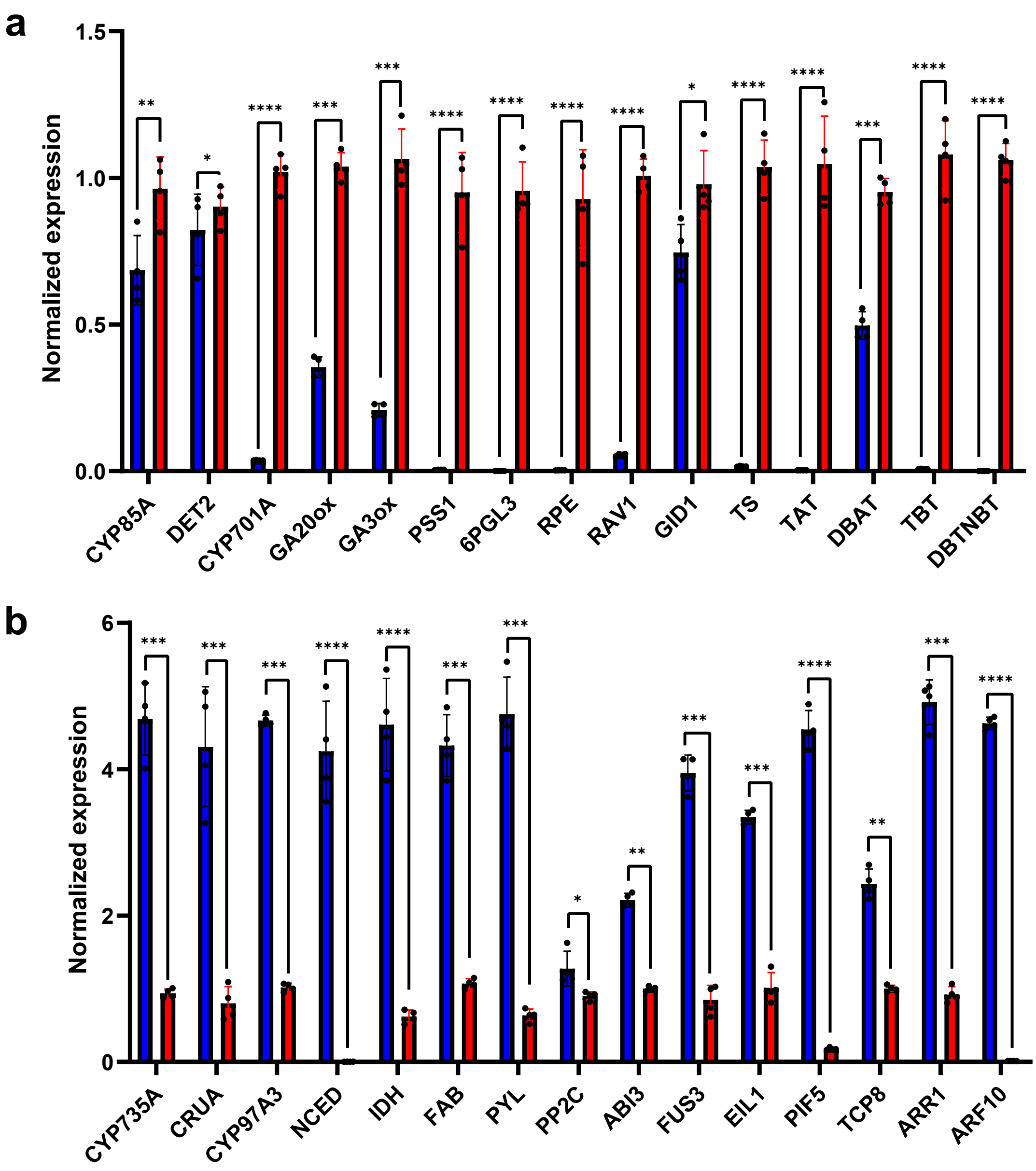

Supplementary Fig. 2. RT-qPCR analyses verify several observed alterations in transcript abundances during Chinese yew seed germination. Each bar represents the average of three biological replicates and three technical replicates (±SD). Student's t-test, P value < 0.0001: ****; 0.0001 < P value < 0.001: ***; 0.001 < P value < 0.01: **; 0.01 < P value < 0.05: *. Bule color denotes dormancy seeds, red color denotes germinating seeds.

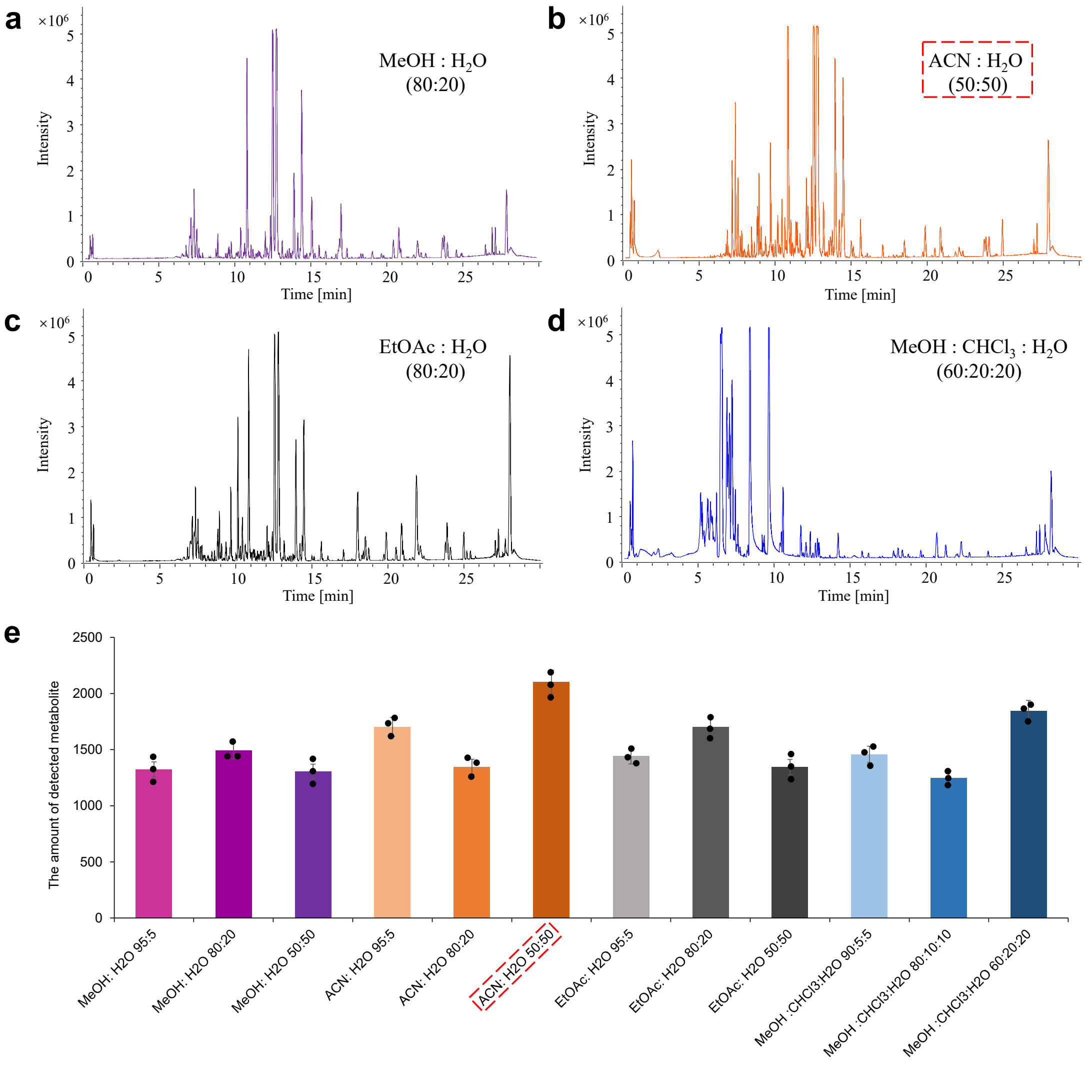

Supplementary Fig. 3. Comparison of chromatographic behavior and extraction efficiency of *T. mairei* seed extracts by different solvents. The upper and middle panels show typically total ion chromatograms, obtained in ESI positive mode, extract with MeOH : H_2O (v/v, 80:20) (**a**), ACN : H_2O (v/v, 50:50) (**b**), EtOAc : H_2O(v/v, 80:20) (**c**) and MeOH : $CHCl_3$: H_2O (v/v, 60:20:20) (**d**). Lower panel (**e**) shows the comparison of the number of detectable metabolites extracted by different solvents.

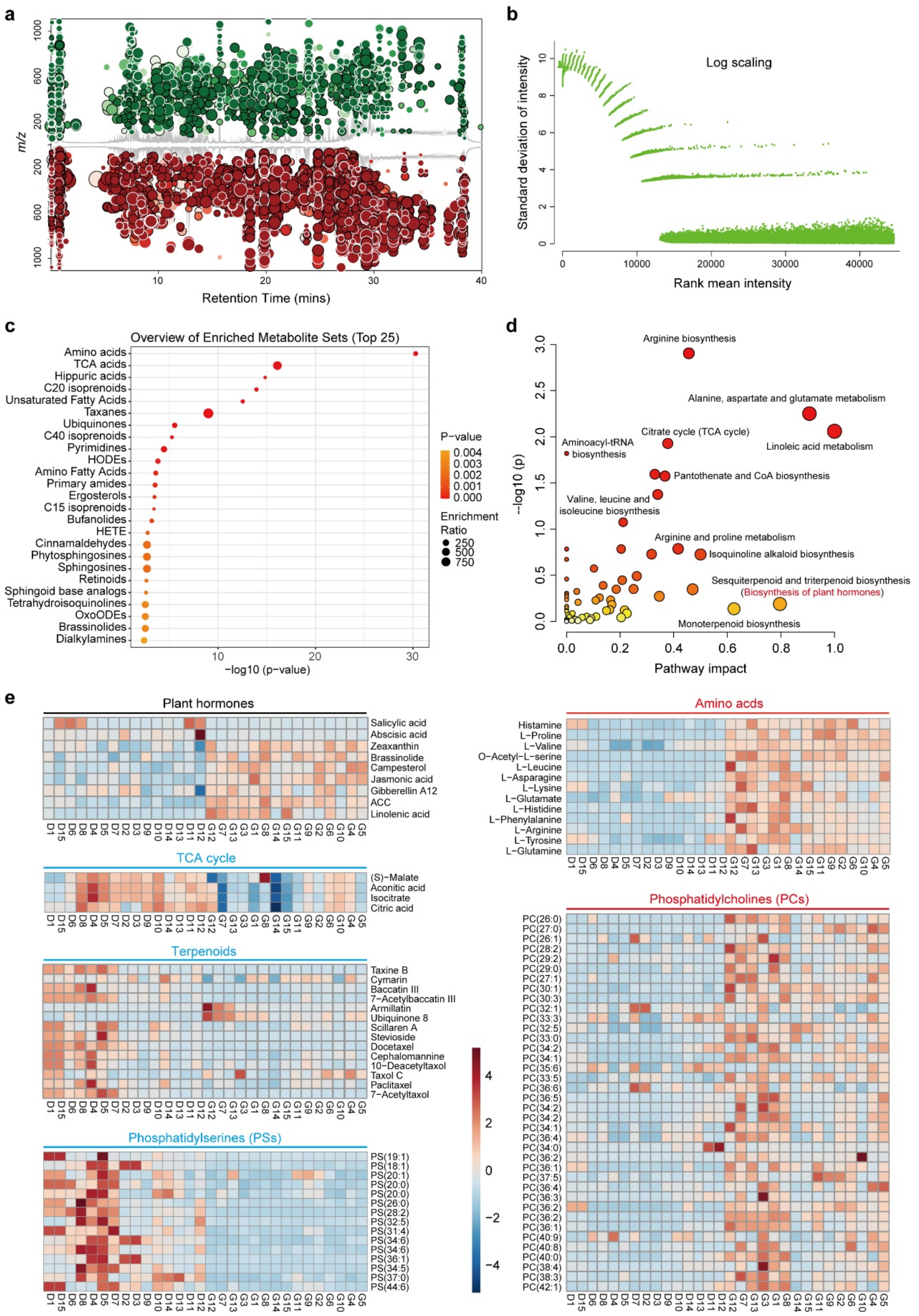

Supplementary Fig. 4. Differentially expressed metabolites between dormant and germinating Chinese yew seeds. a, Cloud plots of the metabolic data of Chinese yew seeds. Data were analysed using XCMS Online, which is freely available at

https://xcmsonline.scripps.edu/. **b**, Scatter plot of rank mean intensity against the standard deviation of intensity in log scaling. **c** and **d**, Enrichment analysis of differentially expressed metabolites. Enriched metabolite sets (top 25) and pathways are shown in **c**) and **d**), respectively. The enrichment ratio was computed using the observed hits/expected hits. **e**, Heatmap of several differentially expressed metabolites between dormant and germinating Chinese yew seeds. Notably, TCA cycle-related metabolites, terpenoids, and phosphatidylserines showed higher expression levels in dormant seeds, whereas amino acids and phosphatidylcholines accumulated to a greater extent in germinating seeds.

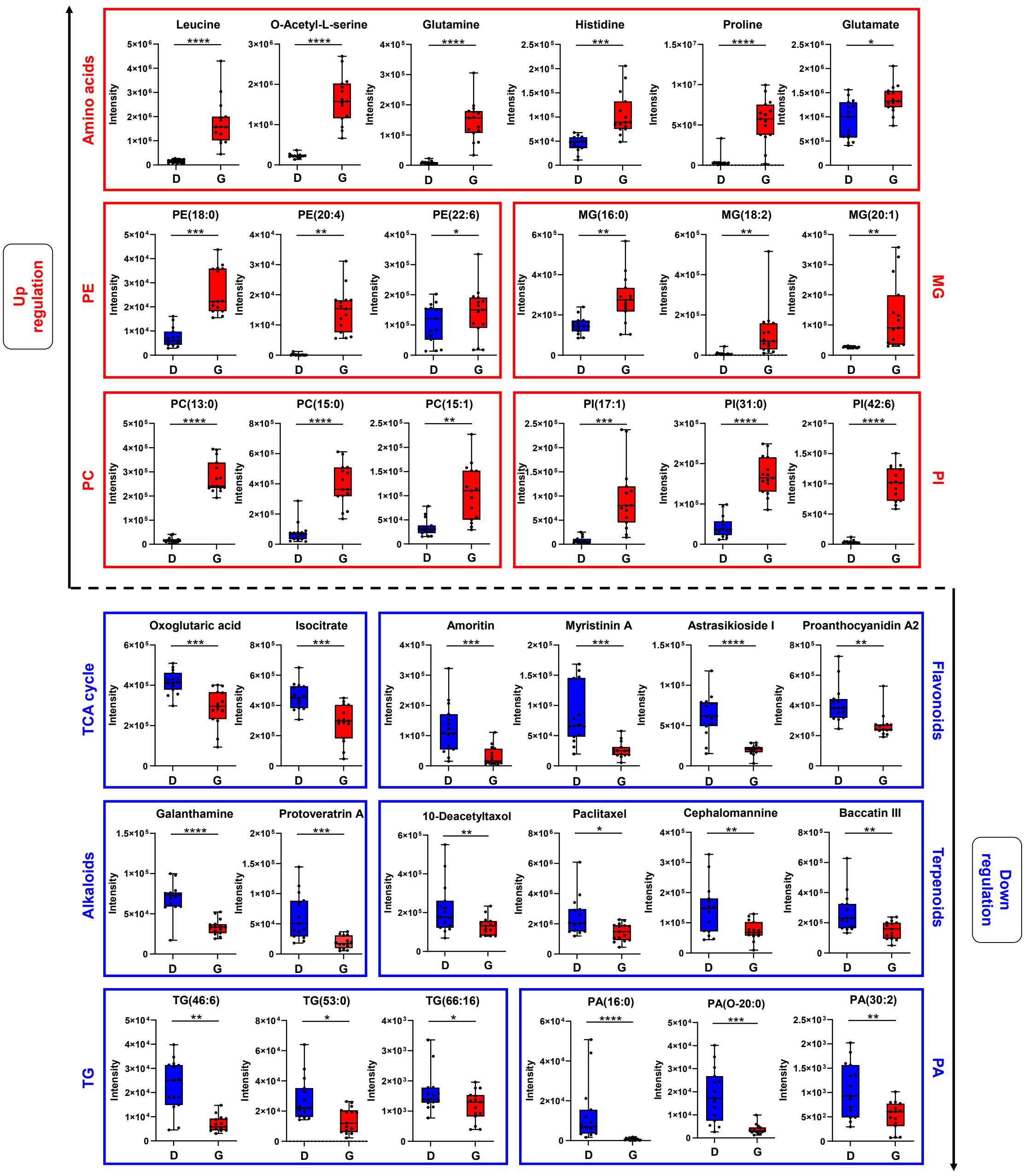

Supplementary Fig. 5. Box plots of metabolites significantly increased (red) and significantly decreased (blue) in germinating Chinese yew seeds detected by UPLC-MS/MS. PE: phosphatidylethanolamine; MG: monoacylglycerol; PC: Phosphocholine; PI: Phosphoinositols; TG: triacylglycerols; PA: phosphatidic acids. D and G represent dormancy group and germination group, respectively. Student's t-test, P value < 0.0001: ****; 0.0001 < P value < 0.001: ***; 0.001 < P value < 0.01: **; 0.01 < P value < 0.05: *.

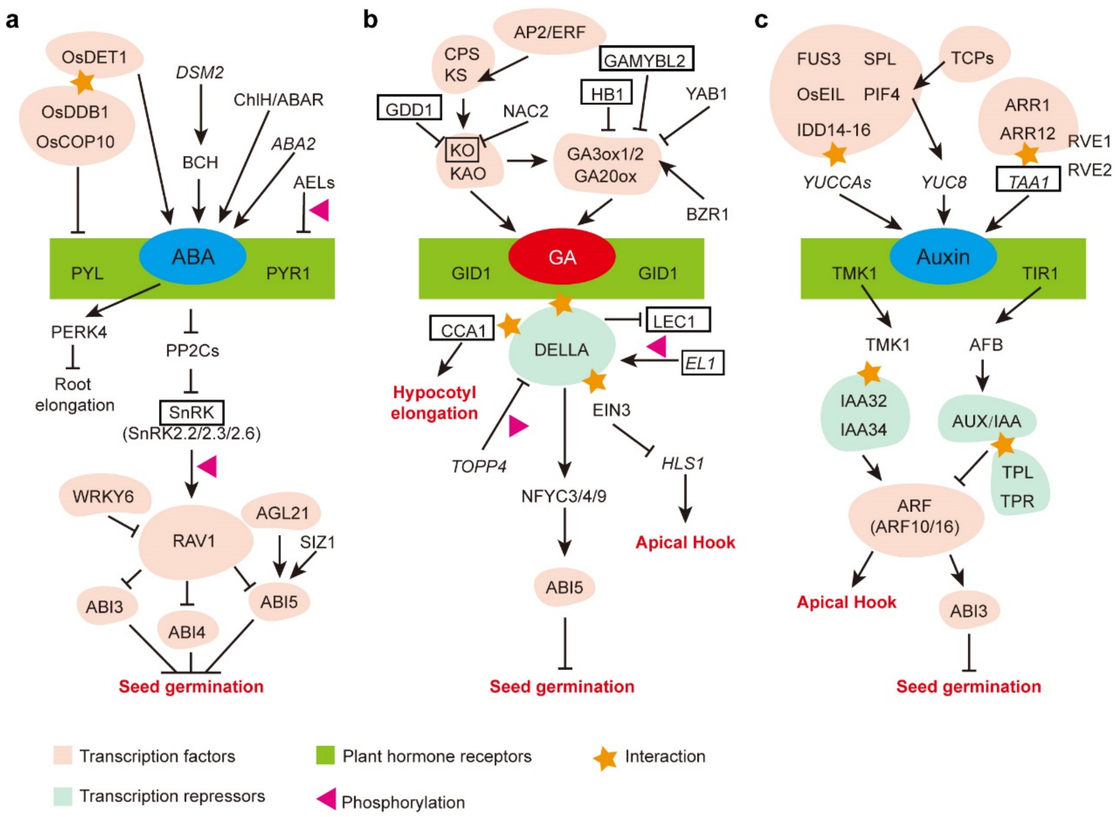

Supplementary Fig. 6. ABA, GA and auxin signaling. Arrows indicate positive regulation and bars indicate negative regulation.

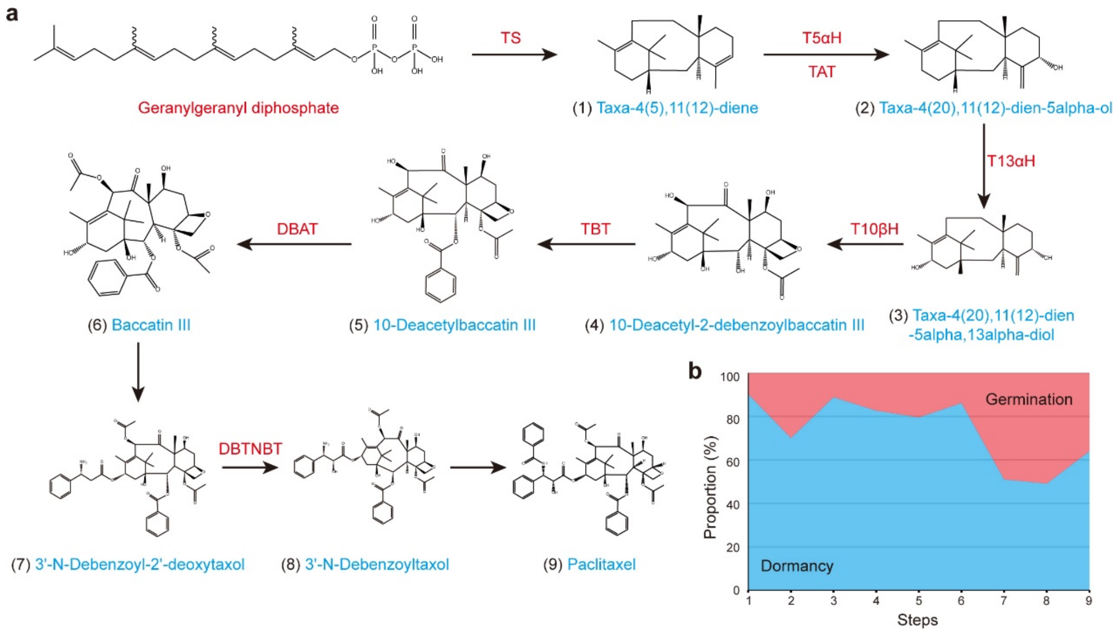

Supplementary Fig. 7. Biosynthesis pathway of paclitaxel in Chinese yew seeds. a, Precursors and intermediates of paclitaxel. TS, taxadiene synthase; T5αH, taxadiene 5-α-hydroxylase; TAT, taxadiene 5-α-ol O-acetyltransferase; T13αH, taxane 13-α-hydroxylase; T10βH, taxane 10-β-hydroxylase; TBT, 2-α-hydroxytaxane-2-O-benzoyltransferase; DBAT, 10-deacetylbaccatinIII 10-O-acetyltransferase; DBTNBT, 3'-N-debenzoyl-2'-deoxytaxol N-benzoyl transferase. **b**, Relative metabolite abundance profiles of paclitaxel biosynthesis precursors and intermediates.

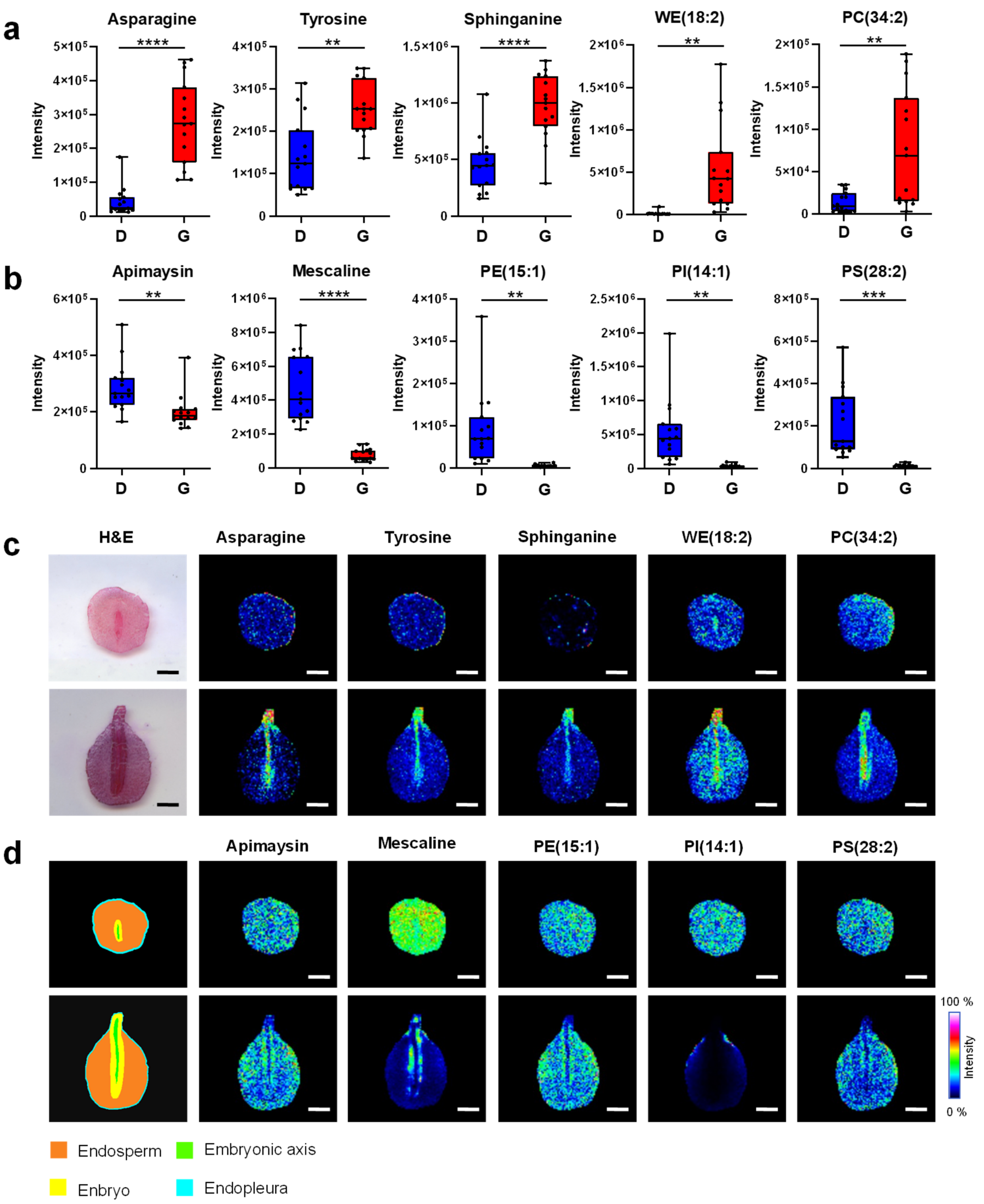

Supplementary Fig. 8. Spatial metabolome difference between dormant and germinating Chinese yew seeds. a, Box plots of concentration significantly increased metabolites in germinating seeds (detected by LC-MS/MS). **b**, Box plots of metabolites with the concentrations significantly decreased in germinating seeds (detected by LC-MS/MS). The upper, middle, and lower lines correspond to the first, second, and third quartiles (25th, 50th, and 75th percentiles), respectively. Student's t-test, P value < 0.0001: ****; 0.0001 < P value < 0.001: ***; 0.001 < P value < 0.01: **; 0.01 < P value < 0.05: *; P value > 0.05: no significant. D and G represent dormancy and germination groups,

respectively. The biological replicate number (n) is 15 for each sample group. **c** and **d**, Spatial distribution of the corresponding metabolites in **a)** and **b)** by MALDI-MSI. MS imaging was performed in the positive-ion mode and acquired at 100 μm spatial resolution. Scale bar, 2 mm.
