# Supplementary material for: Insights from multi-omics integration into seed germination of Taxus chinensis var mairei
Source: Commun Biol. 2023 Sep 11;6:931. doi: 10.1038/s42003-023-05307-x (PMC10495361; doi:10.1038/s42003-023-05307-x)
Supplement: Supplementary file 2 — Description of Additional Supplementary Files [file 42003_2023_5307_MOESM2_ESM.pdf]

Description of Additional Supplementary Files

File name: Supplementary Data 1
Description: Chinese yew seed gene sequence at transcripts level.

File name: Supplementary Data 2
Description: Transcripts annotation of dormancy seed.

File name: Supplementary Data 3
Description: Transcripts annotation of germinating seed.

File name: Supplementary Data 4
Description: Transcription factors prediction.

File name: Supplementary Data 5
Description: Gene expression at transcripts level (FPKM).

File name: Supplementary Data 6
Description: Differentially expressed genes (transcripts level) between dormant and germinating seed.

File name: Supplementary Data 7
Description: GO analysis of DEGs (at transcripts level).

File name: Supplementary Data 8
Description: Peptides detected in Chinese-yew seed.

File name: Supplementary Data 9
Description: Quantitative proteome dataset of Chinese-yew seed.

File name: Supplementary Data 10
Description: Differentially expressed genes (protein level) between dormant and germinating seed.

File name: Supplementary Data 11
Description: Domain and subcellular localization and KEGG pathway analysis of DEGs (at proteins level).

File name: Supplementary Data 12
Description: List of genes expressed both at transcripts and proteins level.

File name: Supplementary Data 13
Description: Ion signal detected by LC-MS.

File name: Supplementary Data 14
Description: Statistical analysis result for LC-MS.

File name: Supplementary Data 15
Description: Metabolites identification of LC-MS.

File name: Supplementary Data 16
Description: KEGG pathway analysis of differentially expressed metabolites.

File name: Supplementary Data 17
Description: Ion signal detected and identification result for MALDI-MS.

File name: Supplementary Data 18
Description: Primes of mRNA used for qRT-PCR analysis.
