# Supplementary material for: Insights from multi-omics integration into seed germination of Taxus chinensis var mairei
Source: Commun Biol. 2023 Sep 11;6:931. doi: 10.1038/s42003-023-05307-x (PMC10495361; doi:10.1038/s42003-023-05307-x)
Supplement: Supplementary file 7 — Reporting Summary [file 42003_2023_5307_MOESM7_ESM.pdf]

nature portfolio

Corresponding author(s): Xiaodong Wang

Last updated by author(s): May 30, 2023

Reporting Summary

Nature Portfolio wishes to improve the reproducibility of the work that we publish. This form provides structure for consistency and transparency in reporting. For further information on Nature Portfolio policies, see our Editorial Policies and the Editorial Policy Checklist.

Statistics

For all statistical analyses, confirm that the following items are present in the figure legend, table legend, main text, or Methods section.

n/a	Confirmed	
☐	☒	The exact sample size (*n*) for each experimental group/condition, given as a discrete number and unit of measurement
☐	☒	A statement on whether measurements were taken from distinct samples or whether the same sample was measured repeatedly
☐	☒	The statistical test(s) used AND whether they are one- or two-sided<br>*Only common tests should be described solely by name; describe more complex techniques in the Methods section.*
☒	☐	A description of all covariates tested
☒	☐	A description of any assumptions or corrections, such as tests of normality and adjustment for multiple comparisons
☐	☒	A full description of the statistical parameters including central tendency (e.g. means) or other basic estimates (e.g. regression coefficient) AND variation (e.g. standard deviation) or associated estimates of uncertainty (e.g. confidence intervals)
☐	☒	For null hypothesis testing, the test statistic (e.g. *F*, *t*, *r*) with confidence intervals, effect sizes, degrees of freedom and *P* value noted<br>*Give P values as exact values whenever suitable.*
☒	☐	For Bayesian analysis, information on the choice of priors and Markov chain Monte Carlo settings
☒	☐	For hierarchical and complex designs, identification of the appropriate level for tests and full reporting of outcomes
☐	☒	Estimates of effect sizes (e.g. Cohen's *d*, Pearson's *r*), indicating how they were calculated

Our web collection on statistics for biologists contains articles on many of the points above.

Software and code

Policy information about availability of computer code

Data collection

RNA-seq raw data were generated by sequencing using the Illumina Hiseq2500 platform.
Proteomic raw data were collected using QExactive Orbitrap HF mass spectrometer; Thermo Fisher Scientific.
Metabolomic raw data were collected using Bruker Impact II quadrupole time-of-flight mass spectrometer; Bruker Daltonics
Mass spectrometry imaging data were generated using Autoflex Speed MALDI-TOF/TOF mass spectrometer; Bruker Daltonics

Data analysis

All softwares and database used in the present study are publicly available and the corresponding software versions were described in detail in the Methods.
1. SOAPnuke (v.1.4.0) https://github.com/BGIflexlab/SOAPnuke
2. Trinity software (v.2.0.6) https://github.com/trinityrnaseq/trinityrnaseq/wiki
3. RSEM http://bowtie-bio.sourceforge.net/Bowtie2/index.shtml
4.TransDecoder (v3.0.1) https://transdecoder.github.io
5. Bowtie2 http://bowtie-bio.sourceforge.net/Bowtie2/index.shtml
6. MASCOT https://www.matrixscience.com/
7. Proteome Discoverer 1.4 Thermo Scientific
8. ProteoWizard MSConvertGUI https://proteowizard.sourceforge.io/download.html
9. XCMS Online https://xcmsonline.scripps.edu/
10. MetaboAnalyst 5.0 https://www.metaboanalyst.ca/
11. LIPID MAPS Structure Database https://www.lipidmaps.org/
12. MASSBANK https://massbank.eu/MassBank/
13. KEGG http://www.genome.jp/kegg
14. GO http://geneontology.org

15. NR ftp://ftp.ncbi.nlm.nih.gov/blast/db
16. NT ftp://ftp.ncbi.nlm.nih.gov/blast/db
17. Pfam http://pfam.xfam.org
18. COG https://www.ncbi.nlm.nih.gov/COG/
19. FlexAnalysis (v.3.4) https://www.bruker.com/zh/services.html
20. FlexImaging (v.4.1) https://www.bruker.com/zh/services.html

For manuscripts utilizing custom algorithms or software that are central to the research but not yet described in published literature, software must be made available to editors and reviewers. We strongly encourage code deposition in a community repository (e.g. GitHub). See the Nature Portfolio guidelines for submitting code & software for further information.

Data

Policy information about availability of data

All manuscripts must include a data availability statement. This statement should provide the following information, where applicable:

- Accession codes, unique identifiers, or web links for publicly available datasets
- A description of any restrictions on data availability
- For clinical datasets or third party data, please ensure that the statement adheres to our policy

RNA-seq files for the transcriptome analysis are available in Supplemental Table 1 (Associated Figures: 1, 2, 3, 4, Extended Figures: 1, 5, 6, and S1). The raw data for the metabolome and mass spectrometry imaging analysis are available upon reasonable request to the corresponding author.

Research involving human participants, their data, or biological material

Policy information about studies with human participants or human data. See also policy information about sex, gender (identity/presentation), and sexual orientation and race, ethnicity and racism.

Reporting on sex and gender	n/a
Reporting on race, ethnicity, or other socially relevant groupings	n/a
Population characteristics	n/a
Recruitment	n/a
Ethics oversight	n/a

Note that full information on the approval of the study protocol must also be provided in the manuscript.

Field-specific reporting

Please select the one below that is the best fit for your research. If you are not sure, read the appropriate sections before making your selection.

☒ Life sciences ☐ Behavioural & social sciences ☐ Ecological, evolutionary & environmental sciences

For a reference copy of the document with all sections, see nature.com/documents/nr-reporting-summary-flat.pdf

Life sciences study design

All studies must disclose on these points even when the disclosure is negative.

Sample size	Sample size was measured in a way that we are able to obtain statistically differences from at least three biological replicates. The initial fifteen biological replicates are typically used for metabolic profiling.
Data exclusions	No data was excluded.
Replication	The majority of the experiments were conducted in at least three independent experiments and were all proven to be highly reproducible.
Randomization	The biological material used in this study were grown in randomized blocks. In addition, the sample analysis was conducted using a randomized design to account for technical variability over time.
Blinding	The samples were not collected blindly. Since all the data that is described is quantified we do not foresee any avoidable bias.

Reporting for specific materials, systems and methods

We require information from authors about some types of materials, experimental systems and methods used in many studies. Here, indicate whether each material, system or method listed is relevant to your study. If you are not sure if a list item applies to your research, read the appropriate section before selecting a response.

Materials & experimental systems

n/a	Involved in the study
☒	☐ Antibodies
☒	☐ Eukaryotic cell lines
☒	☐ Palaeontology and archaeology
☒	☐ Animals and other organisms
☒	☐ Clinical data
☒	☐ Dual use research of concern
☐	☒ Plants

Methods

n/a	Involved in the study
☒	☐ ChIP-seq
☒	☐ Flow cytometry
☒	☐ MRI-based neuroimaging
